# Multi-omics analysis identifies NFIL3 as a hypoxia-associated immune regulator in septic cardiomyopathy

**DOI:** 10.3389/fimmu.2026.1785241

**Published:** 2026-04-30

**Authors:** Haibei Sun, Yuxiao Feng, Rongjiao Shao, Weizhuo Liu, Zhenyu Ren, Xumin Hou, Bin He

**Affiliations:** 1 Department of Critical Care Medicine and Emergency, Shanghai Chest Hospital, Shanghai Jiao Tong University School of Medicine, Shanghai, China; 2 Department of Cardiology, Shanghai Chest Hospital, Shanghai Jiao Tong University School of Medicine, Shanghai, China; 3 Department of Anesthesiology, Huadong Hospital, Fudan University, Shanghai, China

**Keywords:** hypoxia, immune microenvironment, machine learning, Mendelian randomization, septic cardiomyopathy, single-cell RNA sequencing

## Abstract

**Background:**

Sepsis is a life-threatening syndrome caused by a dysregulated host response to infection and is associated with high mortality in intensive care units. Septic cardiomyopathy (SCM) is a frequent and severe complication of sepsis; however, its underlying molecular mechanisms and immune regulatory networks remain incompletely understood.

**Methods:**

Transcriptomic profiling of septic mouse myocardium was performed to characterize hypoxia-associated immune remodeling at both bulk and single-cell levels. Hypoxia-related genes were identified through integrative differential expression and network analyses. These candidate genes were mapped to human peripheral blood transcriptomic datasets for machine learning–based biomarker selection, followed by two-sample Mendelian randomization analysis to assess their causal relevance to sepsis. *In vivo* and *in vitro* experiments were conducted to validate the mechanistic involvement of key genes in septic cardiomyopathy.

**Results:**

Septic myocardium exhibited prominent activation of hypoxia-related signaling accompanied by immune landscape remodeling, characterized by increased macrophage infiltration. NFIL3, TGM2, and SDC4 were identified as key hypoxia-associated hub genes and showed robust diagnostic performance in independent human peripheral blood cohorts. Two-sample Mendelian randomization analysis demonstrated that genetically predicted higher NFIL3 expression was significantly associated with increased sepsis risk. Single-cell analysis revealed predominant enrichment of *Nfil3* in macrophages. Consistently, *in vivo* and *in vitro* experiments confirmed time-dependent upregulation of NFIL3 and HIF-1α in myocardial macrophages during sepsis, with evident spatial colocalization. Functional experiments further demonstrated that NFIL3 negatively regulates macrophage inflammatory responses, at least in part through inhibition of NF-κB signaling.

**Conclusion:**

This study systematically delineates the molecular basis of septic cardiomyopathy, highlighting hypoxia-driven immune dysregulation as a central pathogenic mechanism. As a key hypoxia-responsive immune regulator, NFIL3 may play a critical role in the development of septic cardiac injury. These findings identify novel molecular targets for the early diagnosis and therapeutic intervention of sepsis and its associated myocardial damage.

## Introduction

Sepsis is a life-threatening organ dysfunction caused by a dysregulated host response to infection and is characterized by immune imbalance and a systemic inflammatory reaction. It remains one of the leading causes of mortality among critically ill patients worldwide. The latest consensus definition emphasizes that the core pathophysiology of sepsis lies not in the infectious agent itself, but in infection-induced multi-organ dysfunction driven by maladaptive host responses (1).

The heart is among the most vulnerable target organs affected during sepsis. Epidemiological evidence indicates that approximately 60% of patients with sepsis develop cardiac dysfunction, commonly referred to as septic myocardial injury (2). Septic cardiomyopathy (SCM) is typically characterized by reversible left ventricular systolic and diastolic dysfunction; however, its pathogenesis is highly heterogeneous and involves multiple mechanisms, including excessive inflammation, metabolic dysregulation, and cellular stress responses. Patients with sepsis complicated by myocardial injury exhibit substantially higher mortality, with a 2–3-fold increased risk of death compared with those without cardiac involvement (3, 4). Despite longstanding clinical recognition of SCM, effective targeted therapies remain unavailable, underscoring that its critical molecular regulatory networks have yet to be fully elucidated (5). Consequently, systematic dissection of SCM pathogenesis and identification of key molecular targets with diagnostic and therapeutic potential are of considerable clinical significance.

Accumulating evidence suggests that immune cell infiltration and inflammatory cytokine production are central to the development of SCM, whereas restoration of immune homeostasis can ameliorate myocardial dysfunction (6, 7). Among infiltrating immune populations, macrophages constitute the predominant cell type within the septic heart (8). Growing recognition of macrophage phenotypic and functional heterogeneity has shifted research emphasis from the traditional M1/M2 polarization paradigm toward a more nuanced understanding of subset-specific regulatory programs (9, 10). However, both clinical and experimental studies demonstrate that anti-inflammatory interventions alone fail to substantially reduce SCM-associated mortality, indicating that single-target anti-inflammatory strategies are insufficient to explain or effectively treat SCM. Therefore, investigation of immunometabolic regulatory mechanisms may provide a promising therapeutic avenue for septic myocardial injury (5).

Hypoxia represents a pervasive yet historically underappreciated pathological feature of sepsis. Microcirculatory dysfunction and impaired oxygen delivery subject the myocardium to sustained hypoxic stress. Beyond directly compromising mitochondrial oxidative phosphorylation, hypoxia exacerbates inflammation and tissue injury by activating hypoxia-inducible transcriptional programs, such as hypoxia-inducible factor-1α (HIF-1α), thereby reshaping immune cell function and metabolic phenotypes (11).

NFIL3 (nuclear factor, interleukin-3–regulated; also known as E4BP4) is a transcription factor regulated by multiple signaling pathways, including circadian rhythm–associated networks and interleukin-3 signaling. As a core component of the circadian regulatory system, NFIL3 plays essential roles in the development, differentiation, and functional maintenance of diverse immune cell populations. It has been recognized as a pivotal molecular hub integrating circadian rhythms, immune responses, and metabolic homeostasis (12, 13). Emerging evidence further indicates that NFIL3 not only participates in immune regulation but also governs metabolic reprogramming and cellular stress responses across distinct metabolic contexts, highlighting its pronounced context-dependent functions in different disease states and cell types (14). Under conditions of infection and stress, the functional orientation and biological significance of NFIL3 may shift in response to microenvironmental cues. However, whether and how NFIL3 contributes to septic cardiomyopathy remain largely unexplored.

In summary, hypoxia and immune dysregulation are central pathogenic drivers of septic cardiomyopathy. Nevertheless, systematic investigations into the interplay between hypoxia signaling and immune regulatory networks in the molecular pathogenesis of SCM are still lacking. Advances in high-throughput transcriptomic profiling and single-cell RNA sequencing enable comprehensive characterization of disease-associated molecular landscapes, capturing gene expression heterogeneity across tissues and individual cell populations (15). In parallel, machine learning approaches, owing to their robust feature selection and predictive capacity, provide powerful tools for identifying core disease-associated biomarkers (16). In this study, we integrate multi-omics bioinformatic analyses with machine learning methods to systematically interrogate bulk transcriptomic and single-cell sequencing data from the GEO database (17). In addition, we sought to experimentally determine whether NFIL3 functions as a regulatory node linking hypoxia signaling to macrophage-mediated inflammatory responses in septic cardiomyopathy. This integrative strategy aims to elucidate immune–metabolic interaction mechanisms underlying SCM progression and to establish a theoretical framework for understanding its molecular basis while identifying potential diagnostic targets.

## Materials and methods

### Data sources and acquisition

Multi-omics datasets used in this study were primarily obtained from the Gene Expression Omnibus (GEO) database. Bulk transcriptomic data from two mouse models of septic cardiomyopathy (GSE229925 and GSE267388) were used for discovery analyses. Human peripheral blood transcriptome data from GSE65682 were used for machine learning–based diagnostic biomarker screening, while independent validation was conducted using additional peripheral blood datasets (GSE134347 and GSE66099) as well as a human myocardial tissue dataset (GSE79962). In addition, single-cell RNA sequencing data from mouse septic myocardium (GSE190856) were analyzed to characterize cell type–specific transcriptional features. Hypoxia-related gene sets were obtained from the HALLMARK_HYPOXIA pathway curated in the Molecular Signatures Database (MSigDB). Detailed information on all datasets and gene sets is summarized in **Supplementary Table S1**.

### Differential expression analysis and functional enrichment

All analyses were conducted using R software (version 4.4.1). After batch effect correction, the integrated mouse myocardial transcriptomic datasets (GSE229925 and GSE267388) were subjected to differential expression analysis. The “limma” package was applied to identify differentially expressed genes (DEGs) between sepsis and control groups (18), with thresholds set at an adjusted P value < 0.05 and |log_2_ fold change| > 1. Volcano plots and hierarchical clustering heatmaps were generated using the “ggplot2” and “pheatmap” packages, respectively. Gene set enrichment analysis (GSEA) was performed using the “clusterProfiler” package based on the HALLMARK gene sets curated in MSigDB.

### Immune cell infiltration analysis

Immune cell infiltration was estimated using the CIBERSORTx algorithm with the LM22 signature matrix (19). Transcriptomic deconvolution was performed to infer the relative proportions of immune cell subsets from bulk expression profiles. Data visualization and intergroup comparisons were conducted using the R packages “ggpubr”.

### Core gene screening and interaction network construction

Hypoxia-related differentially expressed genes were defined as the intersection between genes from the HALLMARK_HYPOXIA pathway and myocardial DEGs identified in septic mice. A protein–protein interaction (PPI) network was constructed using the STRING database and visualized in Cytoscape software (version 3.10.4) (20). Key functional modules within the network were identified using the MCODE plugin, and genes within the highest-scoring modules were defined as candidate core genes. Gene Ontology (GO) and Kyoto Encyclopedia of Genes and Genomes (KEGG) pathway enrichment analyses of these candidate genes were conducted using the “clusterProfiler” package (21).

### Machine learning screening and SHAP method

Mouse core genes were converted to their corresponding human orthologs using the “biomaRt” package. Feature selection was conducted on the human peripheral blood training dataset (GSE65682) using three complementary machine learning algorithms: (1) least absolute shrinkage and selection operator (LASSO) regression implemented with the “glmnet” package under 10-fold cross-validation; (2) random forest analysis using the “randomForest” package; and (3) support vector machine recursive feature elimination (SVM-RFE) performed with the “e1071” and “caret” packages under five-fold cross-validation. Genes consistently selected across all three algorithms were defined as key candidate genes. To improve model interpretability, SHapley Additive exPlanations (SHAP) analysis was performed on an XGBoost model constructed using the candidate genes, implemented with the “shapviz” package (22).

### Diagnostic performance validation

The diagnostic performance of key candidate genes and their multi-gene combination models was evaluated in three independent external validation datasets. Receiver operating characteristic (ROC) curves were generated using the R package “pROC,” and the area under the curve (AUC) along with the corresponding 95% confidence interval (CI) was calculated to assess the ability of each model to discriminate sepsis patients from controls.

### Mendelian randomization analysis

To explore potential causal relationships between candidate gene expression and sepsis susceptibility, a two-sample Mendelian randomization (MR) analysis was performed (23). Single-nucleotide polymorphisms (SNPs) strongly associated with candidate genes (*P* < 5 × 10^-8^) were selected as instrumental variables from cis-eQTL data provided by the eQTLGen consortium. Summary statistics for sepsis were obtained from the ieu-b-69 genome-wide association study (GWAS) dataset available through the IEU OpenGWAS platform. MR analyses were conducted using the “TwoSampleMR” R package, with inverse variance weighting (IVW) applied as the primary analytical method and the weighted median approach used as a sensitivity analysis. Instrument strength was evaluated using *F*-statistics (*F* > 10). Heterogeneity and horizontal pleiotropy were assessed using Cochran’s Q test and the MR-Egger intercept test, respectively, to ensure the robustness of causal inference.

### Single-cell transcriptome data analysis

Mouse myocardial single-cell RNA sequencing data (GSE190856) were analyzed using the “Seurat” R package (version 4.4.0) following a standard workflow (24). Cells were subjected to stringent quality control, followed by log-normalization, identification of highly variable genes, data scaling, and principal component analysis (PCA). Cell clusters were annotated based on canonical marker genes curated from published literature and public cell marker databases. Pearson correlation analysis was performed to evaluate the associations between key gene expression and the hypoxia core transcription factor HIF1A, as well as between key gene expression and immune cell infiltration abundance. Correlation matrices were visualized using the “corrplot” package. In addition, hypoxia activity scores at the single-cell level were calculated using the “AUCell” package, and differences across cell types and experimental conditions were statistically compared (25).

### Establishment of the CLP sepsis model

Healthy male C57BL/6J mice (6–8 weeks old) were purchased from SiPeiFu (Beijing) Biotechnology Co., Ltd. (Beijing, China) and housed under specific pathogen-free (SPF) conditions in the animal facility of Shanghai Chest Hospital (Shanghai, China). Sepsis was induced in mice using cecal ligation and puncture (CLP), a well-established model for polymicrobial sepsis and associated organ injury (26). Briefly, mice were fasted for 12 hours with free access to water prior to surgery. Under inhalation anesthesia with isoflurane (2%), a 0.5 cm midline abdominal incision was made. The cecum was exteriorized, ligated at approximately two-thirds of its length distal to the ileocecal valve, and punctured twice with a 21-gauge needle to allow a small amount of fecal content to extrude. The cecum was then returned to the abdominal cavity, and the incision was closed in layers using 4–0 sutures. Postoperatively, 1 mL of sterile phosphate-buffered saline (PBS) was administered subcutaneously to prevent hypovolemic shock. Sham-operated mice underwent laparotomy with cecum exposure and abdominal closure without ligation or puncture. At 24 and 48 hours post-modeling, mice in each group were euthanized by cervical dislocation. Cardiac tissue samples were then collected immediately for subsequent analysis. All animal experiments were conducted in accordance with the guidelines for the care and use of laboratory animals of Shanghai Chest Hospital.

### siRNA transfection and pharmacological treatment

RAW264.7 cells were cultured in DMEM medium (Gibco, 11965-092) supplemented with 10% fetal bovine serum (BIOAGRIO, S1101-100) and 1% penicillin-streptomycin (Absin, abs9244). Cells were transfected with small interfering RNA targeting *Nfil3* (si-Nfil3) or negative control siRNA (si-NC) using a siRNA transfection reagent (GenePharma, G04035) according to the manufacturer’s instructions. After 24–48 hours of transfection, cells were stimulated with LPS (100 ng/mL, Invivogen, tlrl-3pelps) and IFN-γ (20 ng/mL, PeproTech, 300-02) for the indicated time periods. For inhibition of NF-κB signaling, cells were pretreated with BAY 11-7082 (MedChemExpress, HY-13453) prior to stimulation. Knockdown efficiency of *Nfil3* was confirmed by RT-qPCR and Western blot prior to functional experiments.

### Real-time quantitative PCR

RAW264.7 macrophages were stimulated with LPS (100 ng/mL, Invivogen, tlrl-3pelps) plus IFN-γ (20 ng/mL, PeproTech, 300-02) for M1 polarization or with IL-4 (20 ng/mL, PeproTech, P07750) for M2 polarization. After 24 h, total RNA was isolated using RNAiso Plus (Takara, 9109). Complementary DNA (cDNA) was synthesized using the Color Reverse Transcription Kit with gDNA Remover (EZBioscience, A0010CGQ). Quantitative PCR was performed using the 2× Color SYBR Green qPCR Master Mix (ROX2) (EZBioscience, A0012-R2) in a total reaction volume of 10 μL. The amplification protocol consisted of an initial denaturation at 95 °C for 30 seconds, followed by 40 cycles of denaturation at 95 °C for 5 seconds and annealing/extension at 60 °C for 30 seconds. Relative gene expression levels were calculated using the 2^−ΔΔCt^ method and normalized to *Gapdh* as internal reference gene. Primer sequences used for RT-qPCR are listed in **Table 1**.

**Table 1:** Primer sequences used for qPCR.

Gene	Forward primer (5′→3′)	Reverse primer (5′→3′)
*Nfil3*	CTTTCAGGACTACCAGACATCCAA	GATGCAACTTCCGGCTACCA
*Il6*	CCAAGAGGTGAGTGCTTCCC	CTGTTGTTCAACCTCTCTCCCT
*Nppa*	TACAGTGCGGTGTCCAACACAG	TGCTTCCTCAGTCTGCTCACTC
*Nppb*	GAGGTCACTCCTATCCTCTGG	GCCATTTCCTCCGACTTTTCTC
*Gapdh*	AAGTGGTGATGGGCTTCCC	GGCAAATTCAACGGCACAGT

### Western blot analysis

Excised heart tissues and cultured cells were lysed and processed for protein extraction. Briefly, heart tissues were immediately snap-frozen in liquid nitrogen and stored at −80 °C until further processing, while cultured cells were lysed directly on ice. Samples were homogenized in RIPA lysis buffer (Sigma-Aldrich, 89900) supplemented with protease and phosphatase inhibitors (Beyotime, P1046). Total protein concentrations were determined using a bicinchoninic acid (BCA) assay kit (Beyotime Technology, P0009). Equal amounts of protein were separated by 12.5% SDS–PAGE (Epizyme, PG113) and transferred onto 0.45 μm PVDF membranes (Millipore, IPVH00010). Membranes were blocked with 5% non-fat milk for 2 hours at room temperature for non-phospho-specific antibodies, while 5% BSA was used for phospho-specific antibodies to minimize nonspecific binding. Membranes were then incubated overnight at 4 °C with primary antibodies against NFIL3 (Proteintech, 11773-1-AP), HIF-1α (Proteintech, 82989-4-RR), BAX (Cell Signaling Technology, #2772), BCL-2 (Cell Signaling Technology, #3498), phospho-NF-κB p65 (Ser536) (Abmart, PC0982), NF-κB p65 (Proteintech, 10745-1-AP), and GAPDH (Proteintech, 10494-1-AP; Solarbio, K300007RR). After washing with TBST, membranes were incubated with horseradish peroxidase (HRP)–conjugated goat anti-rabbit secondary antibody (Abmart, M21002) for 2 hours at room temperature. Protein bands were visualized using an enhanced chemiluminescence (ECL) detection system (NCM Biotech, P10300), and signal intensities were quantified by densitometric analysis using ImageJ software.

### Triplex immunofluorescence staining with tyramide signal amplification

Cardiac tissues were fixed in 4% paraformaldehyde, embedded in optimal cutting temperature (OCT) compound, and cryosectioned. After air-drying and refixation, tissue sections were permeabilized with 0.1% Triton X-100 and blocked with 10% normal rabbit serum (Servicebio, G1209). Sequential multiplex immunofluorescence staining for F4/80, HIF-1α, and NFIL3 was performed using a TSA system. Each staining cycle consisted of overnight incubation at 4 °C with the corresponding primary antibody (anti-F4/80, Servicebio GB113373; anti-HIF-1α, Servicebio GB151339; anti-NFIL3, Proteintech 11773-1-AP), followed by incubation with an HRP-conjugated goat anti-rabbit secondary antibody (Servicebio, GB23303) and the appropriate fluorophore-labeled tyramide reagent (iF488, iF555, or iF647; Servicebio G1231, G1233, G1232). After each staining cycle, antibody stripping was achieved by heat-mediated antigen retrieval at 95 °C for 15 minutes. Nuclei were counterstained with DAPI, and sections were mounted for imaging. Fluorescence images were acquired using a fluorescence microscope and analyzed with ImageJ software.

### Hematoxylin and eosin staining

Cardiac tissues were fixed, dehydrated through a graded ethanol series, cleared with xylene, and embedded in paraffin. Paraffin-embedded tissues were sectioned into 4–6 μm thick slices using a microtome. Sections were subsequently deparaffinized, rehydrated, and stained with hematoxylin for nuclear visualization and eosin for cytoplasmic staining. After dehydration and mounting with neutral resin, tissue morphology was examined under a light microscope for histopathological evaluation.

### Enzyme-linked immunosorbent assay and cardiac enzyme panel testing

Mouse serum samples were obtained from the retro-orbital venous plexus and processed according to standard procedures. ELISA assays were performed in strict accordance with the manufacturers’ instructions. Absorbance was measured at 450 nm using a microplate reader, and analyte concentrations were calculated based on standard curves. For assessment of myocardial injury, serum levels of lactate dehydrogenase (LDH; Servicebio, G1610) and creatine kinase isoenzyme MB (CK-MB; Servicebio, GM1122) were measured using commercial assay kits.

### Echocardiographic assessment

Transthoracic echocardiography was performed using a high-resolution small animal ultrasound system (Vevo 2100, FUJIFILM VisualSonics). Mice were anesthetized with isoflurane and maintained in the supine position on a temperature-controlled heating pad throughout the procedure. After removal of thoracic hair, ultrasound coupling gel was applied to the chest surface. Parasternal long-axis and short-axis views of the left ventricle were acquired. Left ventricular M-mode images were recorded at the level of the papillary muscles to assess cardiac function, including left ventricular ejection fraction (LVEF), fractional shortening (FS), and cardiac output (CO).

### Statistical analysis

All statistical analyses were performed using the R statistical environment. Data are presented as mean ± standard error of the mean (SEM). Comparisons between two groups were conducted using Student’s *t*-test for normally distributed data or the Mann–Whitney *U* test for non-normally distributed data. Comparisons among multiple groups were performed using one-way analysis of variance (ANOVA), followed by Bonferroni *post hoc* correction when appropriate. Survival analysis was conducted using the Kaplan–Meier method, with differences between groups evaluated by the log-rank test. For Mendelian randomization analyses, multiple complementary methods were applied, with inverse variance IVW serving as the primary approach for causal inference. A two-sided *P* value < 0.05 was considered statistically significant.

## Results

### Sepsis induces transcriptional and immune remodeling in mouse myocardium

**Figure 1:**
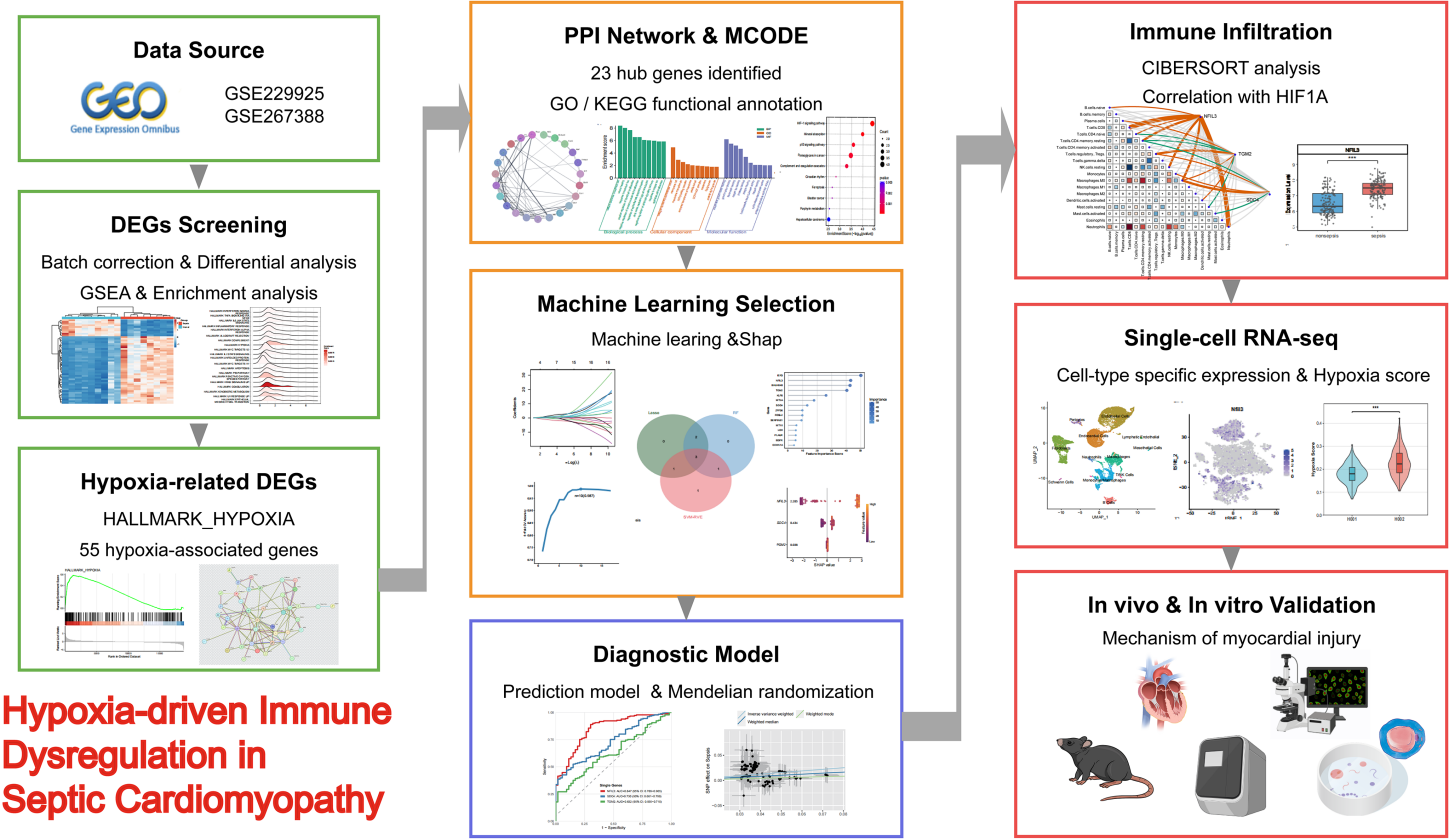
Overview of the study design and analytical workflow.

**Figure 2:**
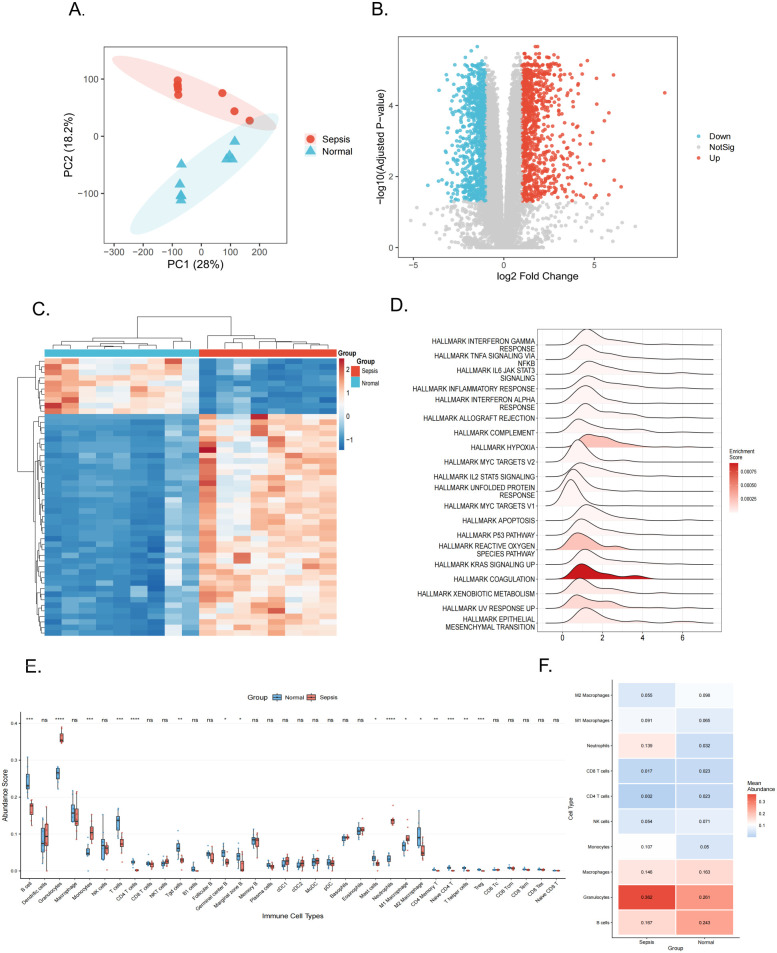
Sepsis induces transcriptomic reprogramming and immune remodeling in mouse myocardium. **(A)** Principal Component Analysis (PCA) plot comparing sepsis vs. normal control samples. **(B)** Volcano plot showing differentially expressed genes between groups (red, upregulated; blue, downregulated). **(C)** Hierarchical clustering heatmap of the top 50 differentially expressed genes between the sepsis and normal control groups. **(D)** Gene set enrichment analysis (GSEA) ridge plot based on HALLMARK metabolism-related gene sets, illustrating metabolic reprogramming between groups. **(E, F)** Immune cell infiltration landscape. Immune cell composition analyzed using the CIBERSORTx deconvolution algorithm. **(E)** Box plot comparing infiltration proportions for each immune cell subset between the two groups. **(F)** Heatmap visualizing the relative proportions of infiltrating immune cell subsets across samples, with color intensity indicating normalized infiltration levels. Numerical values are displayed within each cell. (****P < 0.0001, ***P < 0.001, **P < 0.01, *P < 0.05; ns, no significant difference).

The study workflow is outlined in **Figure 1**. To systematically reveal the molecular characteristics of sepsis, we conducted an integrated analysis of myocardial transcriptome data from septic mice. Principal Component Analysis (PCA) showed distinct separation of overall gene expression patterns between the two groups (**Figure 2A**). Differential expression analysis identified numerous genes that were significantly upregulated or downregulated in sepsis (**Figure 2B**), and the top 50 most altered genes were clearly differentiated between the groups in a heatmap (**Figure 2C**).

To investigate functional alterations, we performed gene set enrichment analysis (GSEA) focusing on metabolism-related pathways within the HALLMARK gene sets, which revealed extensive metabolic reprogramming in myocardial tissue during sepsis (**Figure 2D**). Immune cell composition in cardiac tissue from a mouse model of septic cardiomyopathy was then analyzed using the CIBERSORTx deconvolution algorithm. The immune cell infiltration landscape was summarized in a stacked bar chart, which illustrated proportional changes in all major cell types between septic and sham controls (**Supplementary Figure S1**). Quantitative analysis revealed significant alterations in the infiltration proportions of several immune cell subsets (**Figure 2E**). A heatmap further visualized the remodeling of the cardiac immune microenvironment, highlighting prominent features of immune remodeling, including a marked increase in granulocyte infiltration and a concomitant reduction in M2 macrophages (**Figure 2F**).

Focusing on macrophages, we observed a marked shift in polarization status within the septic myocardium. Specifically, the proportion of M1 macrophages was significantly increased (*P* < 0.05), whereas the proportion of M2 macrophages was significantly reduced (*P* < 0.05), resulting in a substantially elevated M1/M2 ratio (**Supplementary Figure S2**, *P* = 1.6 × 10^-5^).

### Hypoxia-related genes form a core regulatory network in septic myocardium

**Figure 3:**
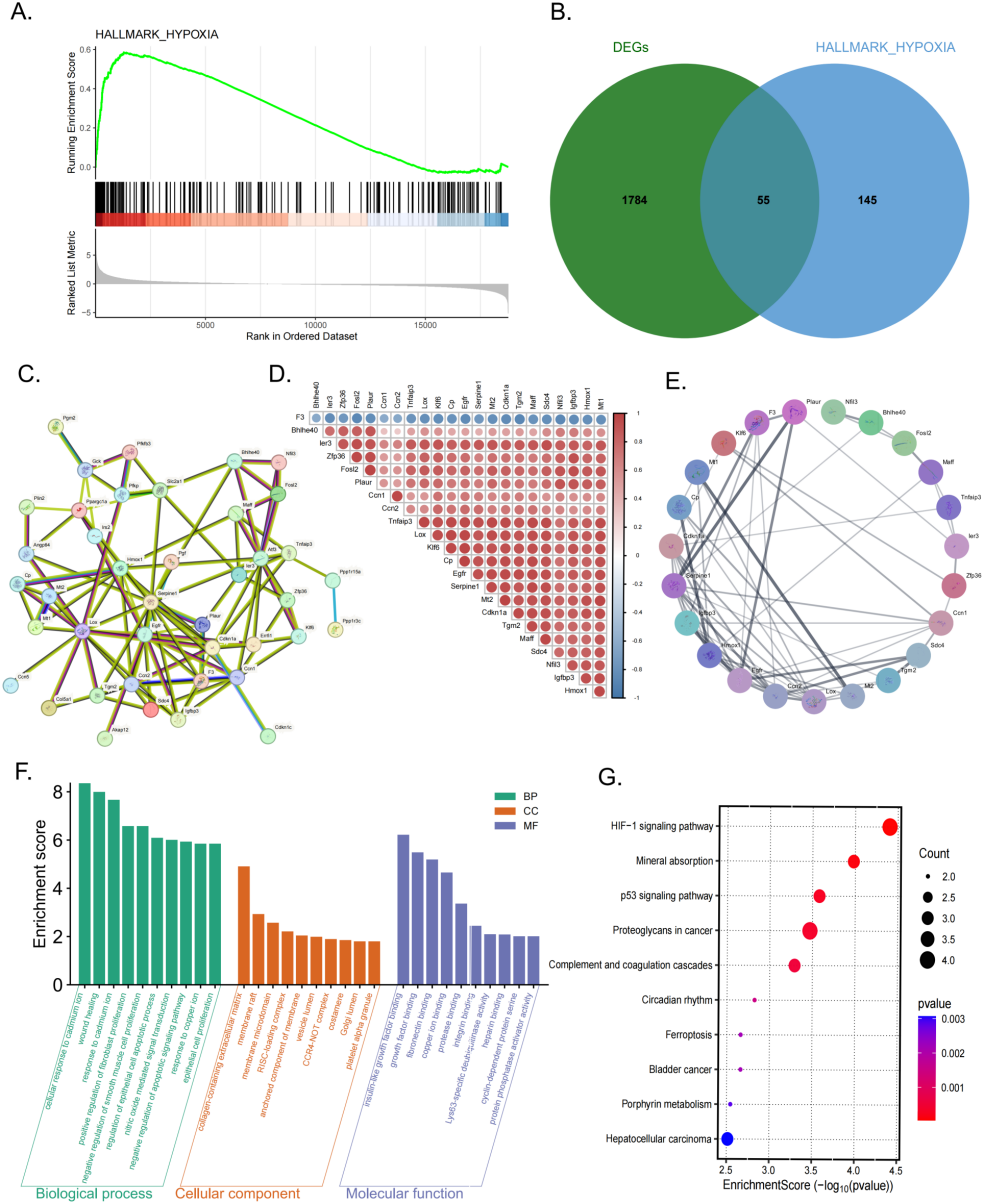
Identification of hypoxia-related core genes and functional pathways in septic myocardium. **(A)** GSEA enrichment plot for the HALLMARK_HYPOXIA pathway. **(B)** Venn diagram showing the overlap between DEGs and the HALLMARK_HYPOXIA gene set. **(C)** Protein–protein interaction (PPI) network constructed based on the 55 hypoxia-related DEGs. **(D)** Heatmap showing expression level correlations of the aforementioned 23 core genes across samples. **(E)** Highest-scoring core module identified from the PPI network via the MCODE algorithm, comprising 23 hub genes. **(F)** Gene Ontology (GO) enrichment analysis diagram for the 23 core genes. **(G)** KEGG pathway enrichment bubble plot for the 23 core genes.

GSEA analysis demonstrated significant enrichment of the HALLMARK_HYPOXIA pathway in septic myocardium compared with controls (**Figure 3A**). To further characterize hypoxia-associated transcriptional changes, differentially expressed genes were intersected with the HALLMARK_HYPOXIA gene set, yielding 55 shared hypoxia-related DEGs (**Figure 3B**). A protein–protein interaction (PPI) network was constructed based on these genes (**Figure 3C**), and the MCODE algorithm identified the highest-scoring core module comprising 23 hub genes (**Figure 3E**). Correlation analysis revealed strong co-expression patterns among these 23 core genes across samples (**Figure 3D**). Functional enrichment analysis indicated that these genes were significantly associated with biological processes including wound healing and nitric oxide–mediated signaling (**Figure 3F**). KEGG pathway analysis further revealed enrichment in hypoxia-related and stress-response pathways, notably the HIF-1 and p53 signaling pathways (**Figure 3G**). To translate these findings into clinically relevant biomarkers, we next applied machine learning to human sepsis transcriptomic datasets.

### Integrated machine learning analysis identifies hypoxia-associated diagnostic biomarkers

**Figure 4:**
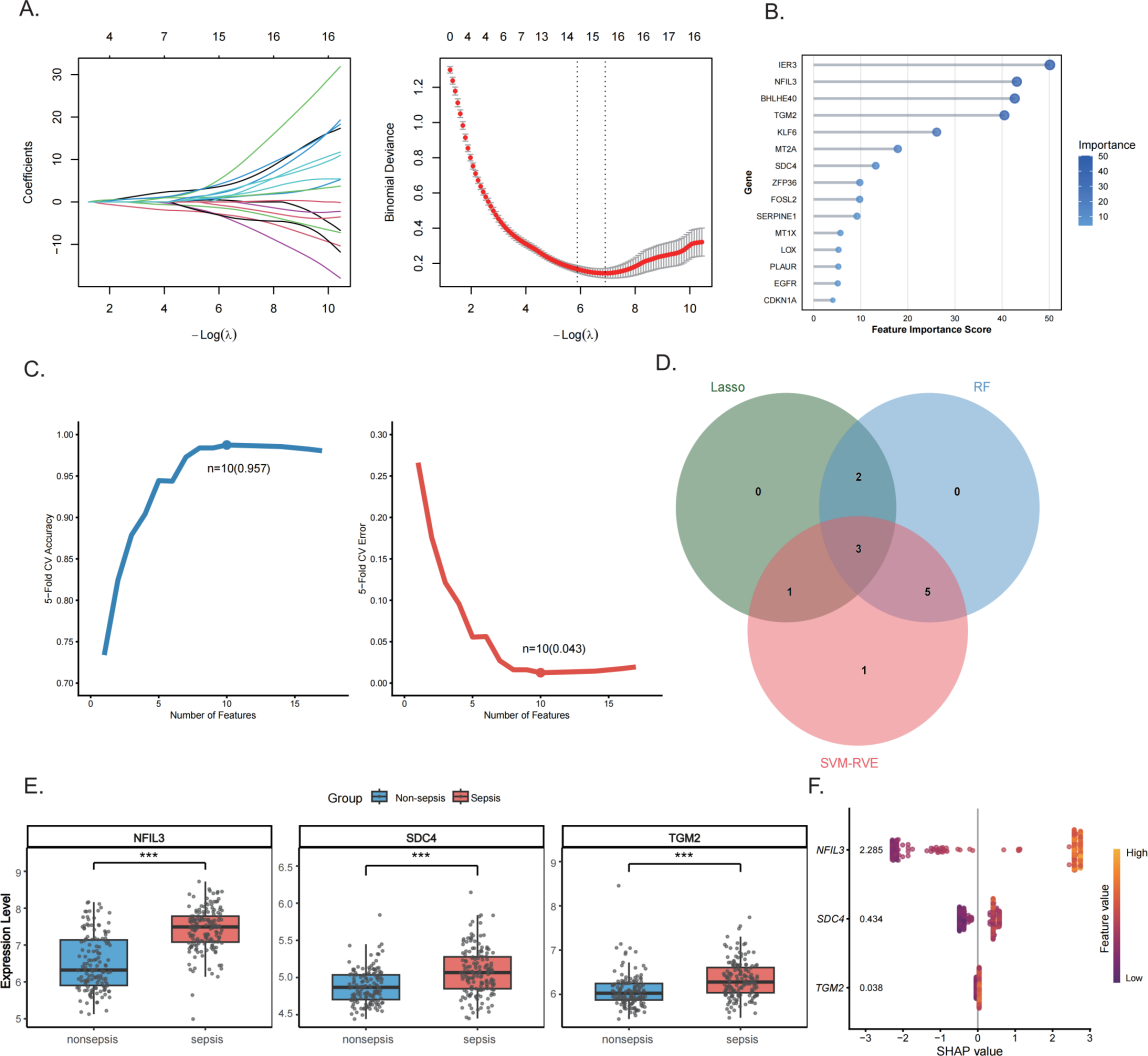
Identification of key diagnostic biomarkers for sepsis in human peripheral blood using machine learning and SHAP analysis. **(A)** Coefficient path plot of LASSO regression as a function of the penalty parameter (−logλ), with the optimal λ determined by 10-fold cross-validation. **(B)** Ranking of the top 15 most important genes in the Random Forest (RF) algorithm. **(C)** Cross-validation results of Support Vector Machine Recursive Feature Elimination (SVM-RFE). **(D)** Venn diagram of three pivotal genes obtained from the intersection of SVM-RFE, RF, and LASSO algorithm results. **(E)** Expression level comparison of three key genes (NFIL3, TGM2, SDC4) in peripheral blood between sepsis patients and healthy individuals (***P < 0.001). **(F)** SHAP (Shapley Additive Explanations) interpretability analysis swarm plot illustrating contribution patterns of three key genes in the XGBoost sepsis diagnostic model.

To translate hypoxia-related core genes identified in mouse myocardium into clinically relevant biomarkers, mouse genes were mapped to their human orthologs using the biomaRt package based on Ensembl annotations. Feature selection was then performed on human peripheral blood transcriptomic data using three machine learning algorithms. LASSO regression identified the optimal penalty parameter through 10-fold cross-validation (**Figure 4A**). For random forest analysis, model tuning was performed based on the OOB error rate (**Supplementary Figure S3**), and genes were ranked by importance scores (**Figure 4B**). SVM-RFE achieved optimal performance with 10 features and a cross-validation accuracy of 0.957 (**Figure 4C**). ROC curve analyses confirmed the diagnostic performance of all three models (**Supplementary Figures S4A–C**). Intersection analysis of the three algorithms identified three shared candidate biomarkers: NFIL3, TGM2, and SDC4 (**Figure 4D**). Expression analysis revealed that all three genes were significantly upregulated in the peripheral blood of sepsis patients compared with healthy controls (NFIL3: 7.423 ± 0.581 vs. 6.514 ± 0.751; SDC4: 5.08 ± 0.31 vs. 4.881 ± 0.228; TGM2: 6.335 ± 0.415 vs. 6.082 ± 0.379; all *P* < 0.001), with NFIL3 exhibiting the most pronounced expression difference (**Figure 4E**).

### NFIL3 exhibits superior diagnostic performance among hypoxia-associated genes

**Figure 5:**
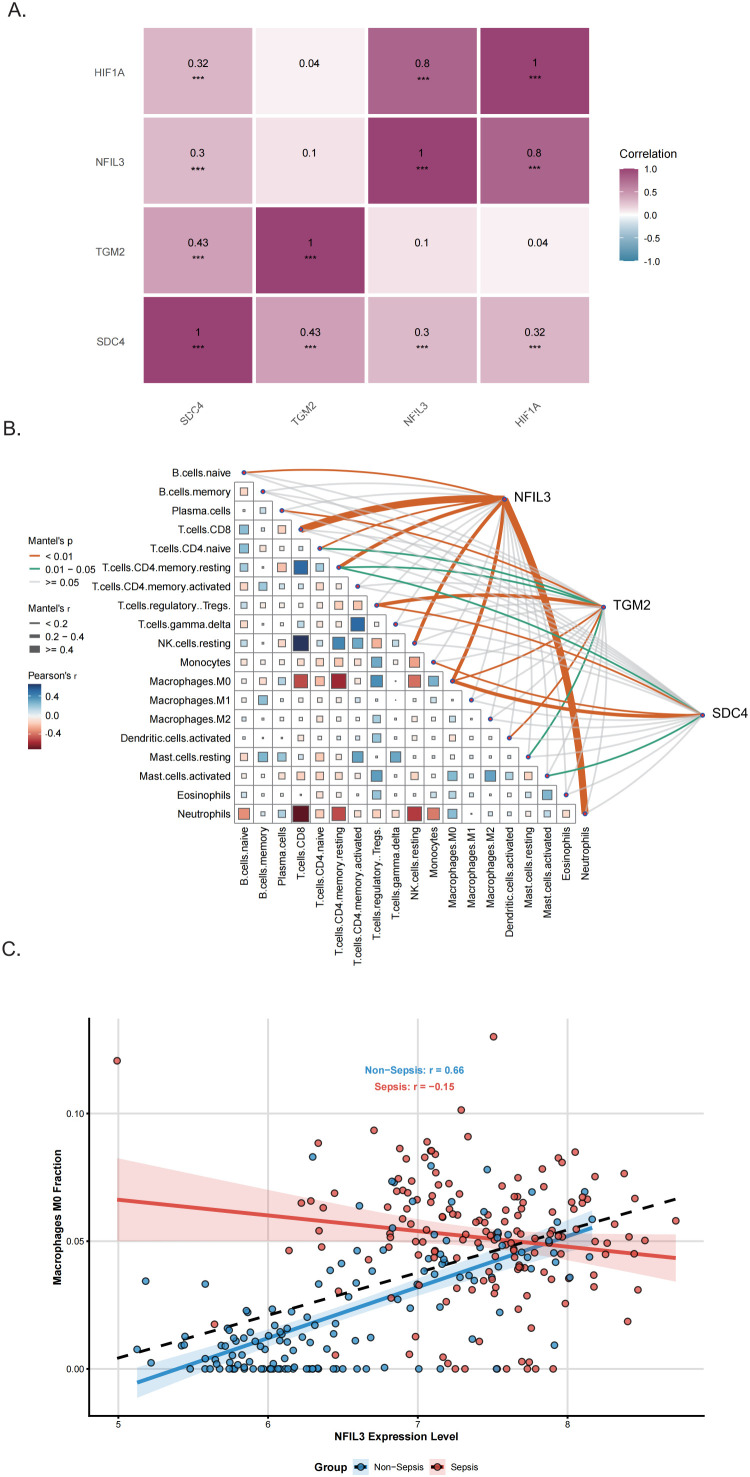
NFIL3 is associated with hypoxia signaling and immune cell infiltration in sepsis. **(A)** Heatmap showing Pearson correlations between key gene expression levels (HIF1A, NFIL3, TGM2, and SDC4). Color intensity indicates the magnitude of the correlation coefficient (r), with exact r values presented in each cell ( ***P < 0.001). **(B)** Correlations between the abundance of specific immune cell types and the expression levels of NFIL3, TGM2, and SDC4. **(C)** Scatter plot comparing NFIL3 expression in M0 macrophages between septic and non-septic groups.

SHAP analysis further demonstrated that NFIL3 contributed the highest feature importance in the XGBoost diagnostic model (mean absolute SHAP value = 2.285), indicating its dominant role in model prediction (**Figure 4F**). Correlation analysis demonstrated significant positive associations between the expression of NFIL3, SDC4, and TGM2 and the hypoxia-related transcription factor HIF1A (**Figure 5A**), suggesting that these genes are embedded within the hypoxia-responsive transcriptional network activated during sepsis. Immune cell infiltration analysis further revealed that the expression levels of NFIL3, SDC4, and TGM2 were significantly correlated with the abundance of multiple immune cell subsets (**Figure 5B**), indicating a potential link between hypoxia-driven transcriptional regulation and immune remodeling in septic cardiomyopathy. Differential expression patterns of these genes across immune cell populations under septic and non-septic conditions are presented in **Supplementary Figure S5**. Notably, NFIL3 expression was markedly elevated in macrophages from septic patients compared with non-septic controls (**Figure 5C**), highlighting macrophages as a key cellular compartment associated with NFIL3 dysregulation during sepsis. Collectively, these findings indicate that NFIL3, together with SDC4 and TGM2, is closely associated with hypoxia-related transcriptional activity and immune cell infiltration patterns, particularly within macrophage-, neutrophil-, and T cell–related compartments.

**Figure 6:**
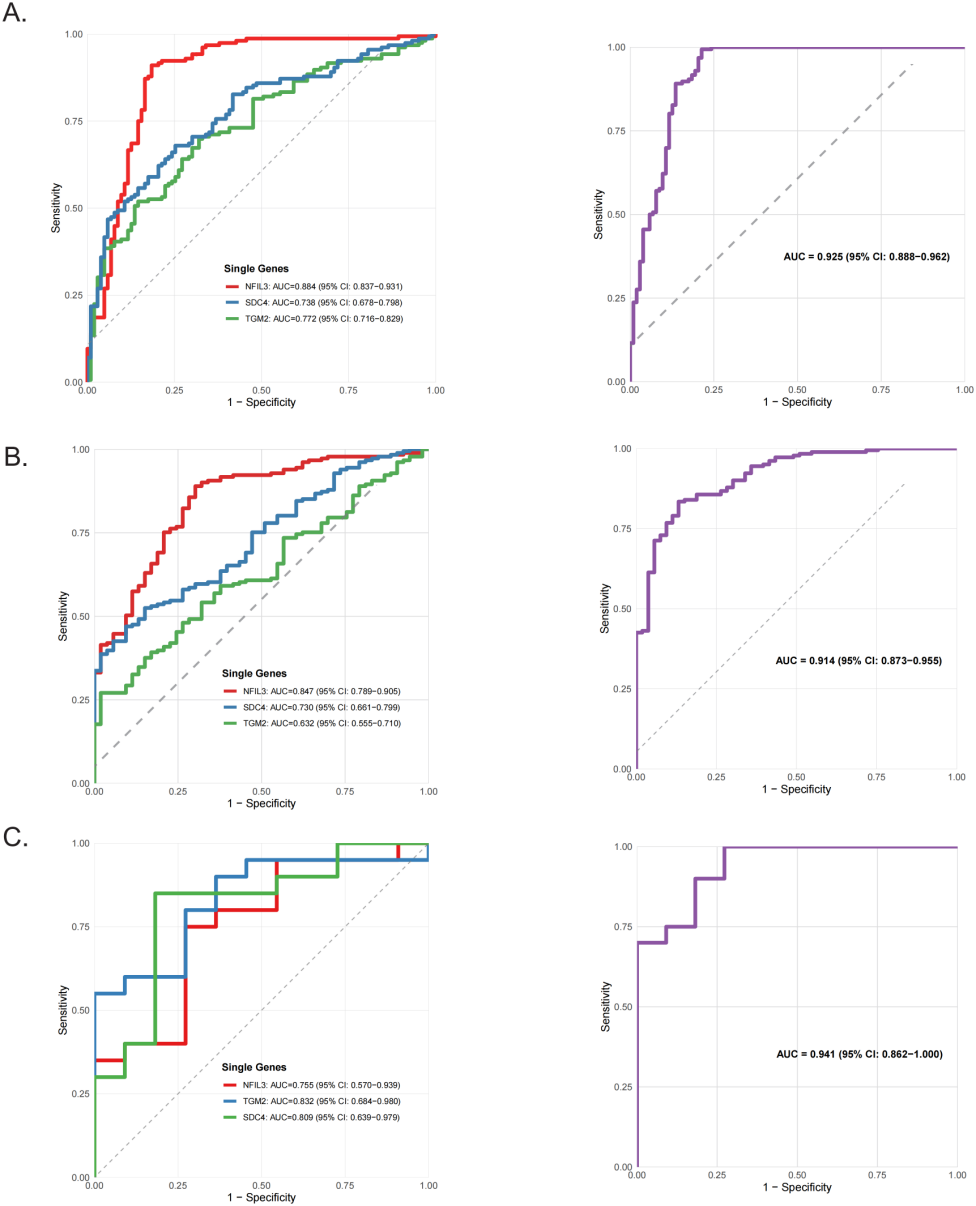
Single-gene and multi-gene models demonstrate robust diagnostic performance across sepsis cohorts. **(A–C)** Receiver operating characteristic **(ROC)** curves of single-gene models (NFIL3, TGM2, and SDC4) and a multi-gene combination model in sepsis peripheral blood datasets GSE134347 **(A)**, GSE66099 **(B)**, and myocardial tissue dataset GSE79962 **(C)**.

To independently evaluate the diagnostic potential of these hypoxia-associated immune genes, validation analyses were performed across three independent sepsis transcriptomic datasets. In the two peripheral blood datasets (GSE134347 and GSE66099), all three genes exhibited robust diagnostic performance. Among them, NFIL3 consistently demonstrated the highest diagnostic accuracy (AUC = 0.884 and 0.847, respectively; **Figures 6A, B**). Integration of NFIL3, SDC4, and TGM2 into a composite diagnostic model further improved discriminatory performance, yielding AUCs of 0.925 (95% CI: 0.888–0.962) and 0.914 (95% CI: 0.873–0.955), respectively. Consistently, in the sepsis myocardial tissue dataset (GSE79962), the combined model achieved superior diagnostic performance (AUC = 0.941, 95% CI: 0.862–1.000; **Figure 6C**), supporting the robustness and cross-tissue applicability of the NFIL3-centered diagnostic signature.

### Genetic evidence links NFIL3 expression to sepsis susceptibility

**Figure 7:**
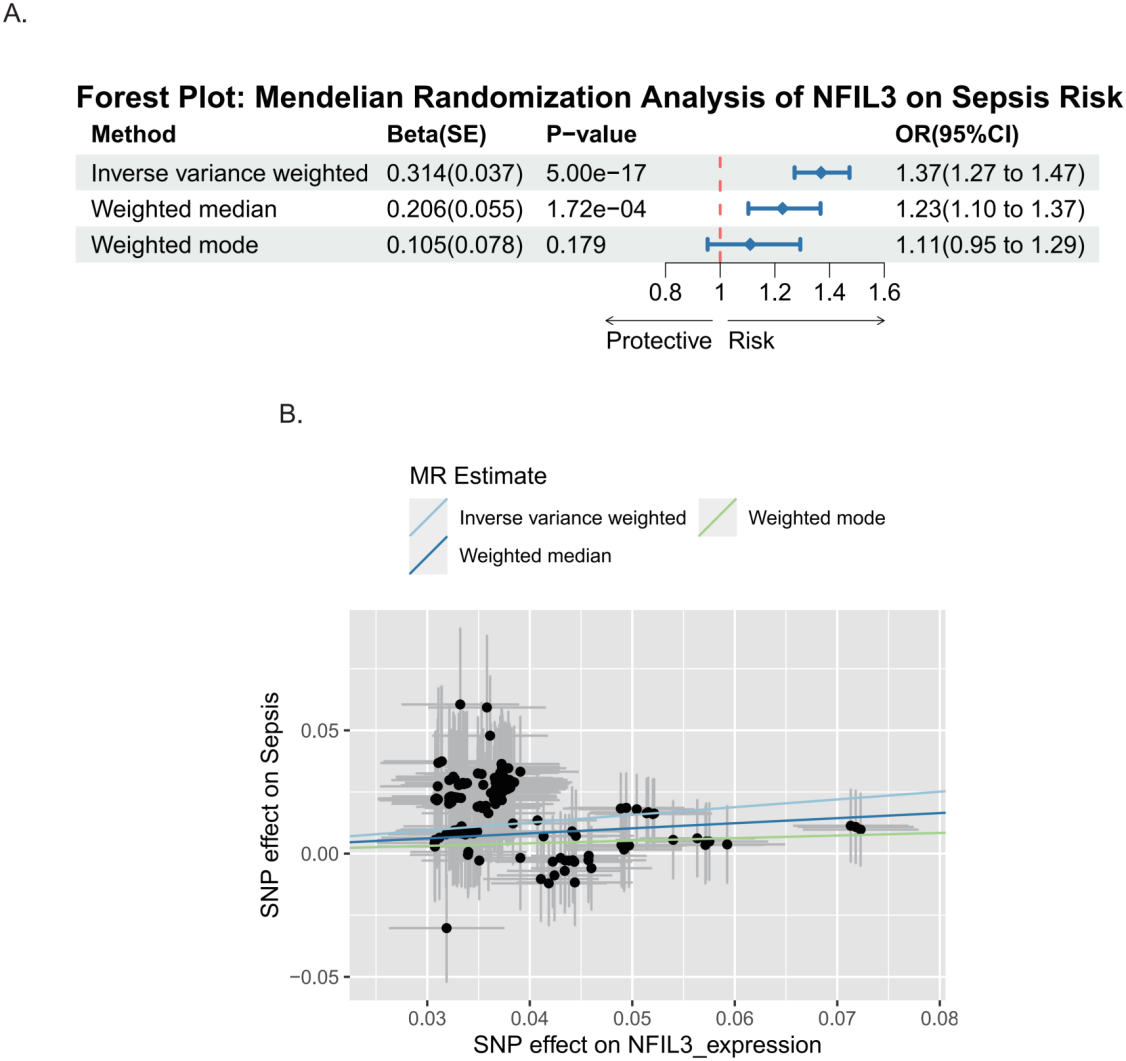
Mendelian randomization analysis of NFIL3 in sepsis. **(A)** Forest plot showing Mendelian randomization estimates for the association between genetically predicted NFIL3 expression and sepsis risk. **(B)** Scatter plot illustrating the associations between single nucleotide polymorphism (SNP)–NFIL3 expression and SNP–sepsis risk.

To further explore potential genetic associations between candidate genes and sepsis susceptibility, a two-sample Mendelian randomization analysis was conducted using expression-associated SNPs as instrumental variables. The results demonstrated that genetically predicted higher NFIL3 expression was significantly associated with an increased risk of sepsis, as estimated by the inverse variance weighted (IVW) method (odds ratio [OR] = 1.369, 95% CI: 1.272–1.473, *P* = 5.00 × 10^-^¹^7^). Consistent effect directions were observed using the weighted median approach (OR = 1.228, 95% CI: 1.103–1.367, *P* = 1.72 × 10^-4^) (**Figures 7A, B**). Sensitivity analyses revealed no significant heterogeneity among instrumental variables (Cochran’s Q *P* > 0.05), and 89.8% of SNPs exhibited concordant effect directions, supporting the robustness of the causal inference. In contrast, Mendelian randomization analyses for TGM2 and SDC4 did not reach conventional significance thresholds and showed limited consistency across methods, providing insufficient evidence for a causal association with sepsis risk. Taken together with multi-cohort diagnostic validation results, these findings prioritize NFIL3 as the most robust candidate gene for subsequent experimental validation and mechanistic investigation.

### Single-cell and experimental validation reveal macrophage-enriched *Nfil3* upregulation in septic cardiomyopathy

**Figure 8:**
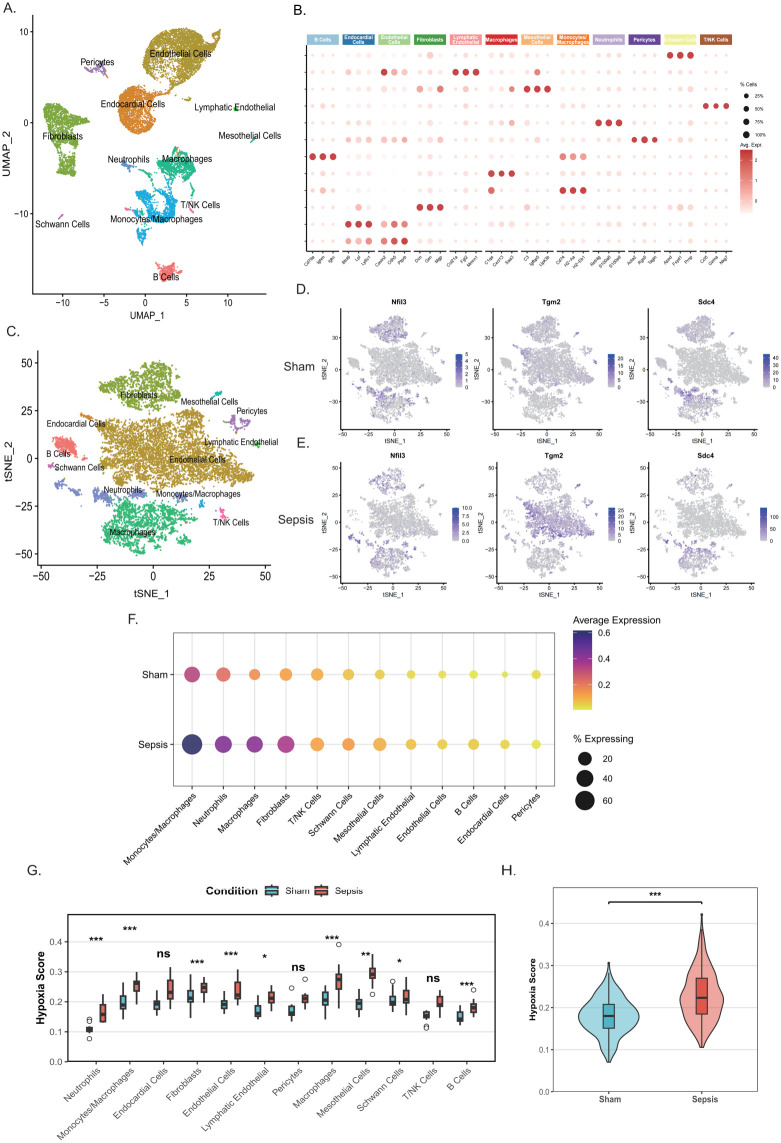
Single-cell analysis reveals cell type–specific hypoxia-associated gene expression in septic myocardium. **(A)** UMAP visualization of integrated single-cell transcriptomic profiles from sham and septic myocardium. **(B)** Dot plots showing the top three differentially expressed marker genes used to annotate major cell clusters. **(C)** t-SNE visualization of the single-cell dataset. **(D, E)** t-SNE plots showing the expression distribution of *Nfil3*, *Tgm2*, and *Sdc4* in the sham group **(D)** and the septic cardiomyopathy group **(E)**. **(F)** Dot plot showing the expression of *Nfil3* across different cell types in the sham and septic cardiomyopathy groups. **(G)** Hypoxia activity scores for each cell cluster calculated using the AUCell algorithm. Asterisks indicate significance after FDR correction (***FDR < 0.001, **FDR < 0.01, *FDR < 0.05; ns, not significant). **(H)** Comparison of overall hypoxia activity between sham and septic cardiomyopathy groups (FDR < 0.001).

To validate hypoxia-related transcriptional responses and key gene expression patterns at the cellular level, we analyzed a public single-cell RNA sequencing dataset of mouse septic cardiomyopathy (GSE190856). Unsupervised clustering combined with dimensionality reduction (UMAP and t-SNE) identified major cardiac and immune cell populations, including cardiomyocytes, fibroblasts, endothelial cells, and macrophages (**Figures 8A, C**), with cluster identities confirmed using canonical marker genes (**Figure 8B**). Comparative analysis revealed that *Nfil3*, *Tgm2*, and *Sdc4* were significantly upregulated in the septic cardiomyopathy group and displayed distinct cell type–specific expression patterns. Notably, *Nfil3* exhibited preferential enrichment in myeloid monocytes/macrophages under basal conditions, with further elevation in septic myocardium. In contrast, *Sdc4* and *Tgm2* showed predominant enrichment in neutrophils and endothelial cells, respectively (**Figures 8D, E**). Consistently, dot plot analysis further demonstrated increased *Nfil3* expression and proportion of expressing cells across multiple cell types, with prominent enrichment in macrophage populations in septic cardiomyopathy (**Figure 8F**). To quantitatively assess hypoxic activity at the single-cell level, hypoxia scores were calculated using the AUCell algorithm. A widespread and cell-specific hypoxic response was observed in septic myocardium, with significantly increased hypoxia activity in endothelial cells (fold change = 1.31), neutrophils (fold change = 1.60), macrophages (fold change = 1.34), and B cells (fold change = 1.54) compared with sham controls (FDR < 0.05; **Figure 8G**). Intergroup comparison further confirmed a marked elevation of overall myocardial hypoxia activity in the septic cardiomyopathy group (**Figure 8H**, FDR < 0.001), indicating coordinated participation of immune and structural cells in hypoxic microenvironment remodeling during septic cardiomyopathy.

### Macrophage subset analysis identifies Nfil3 enrichment in inflammatory and reparative populations

**Figure 9:**
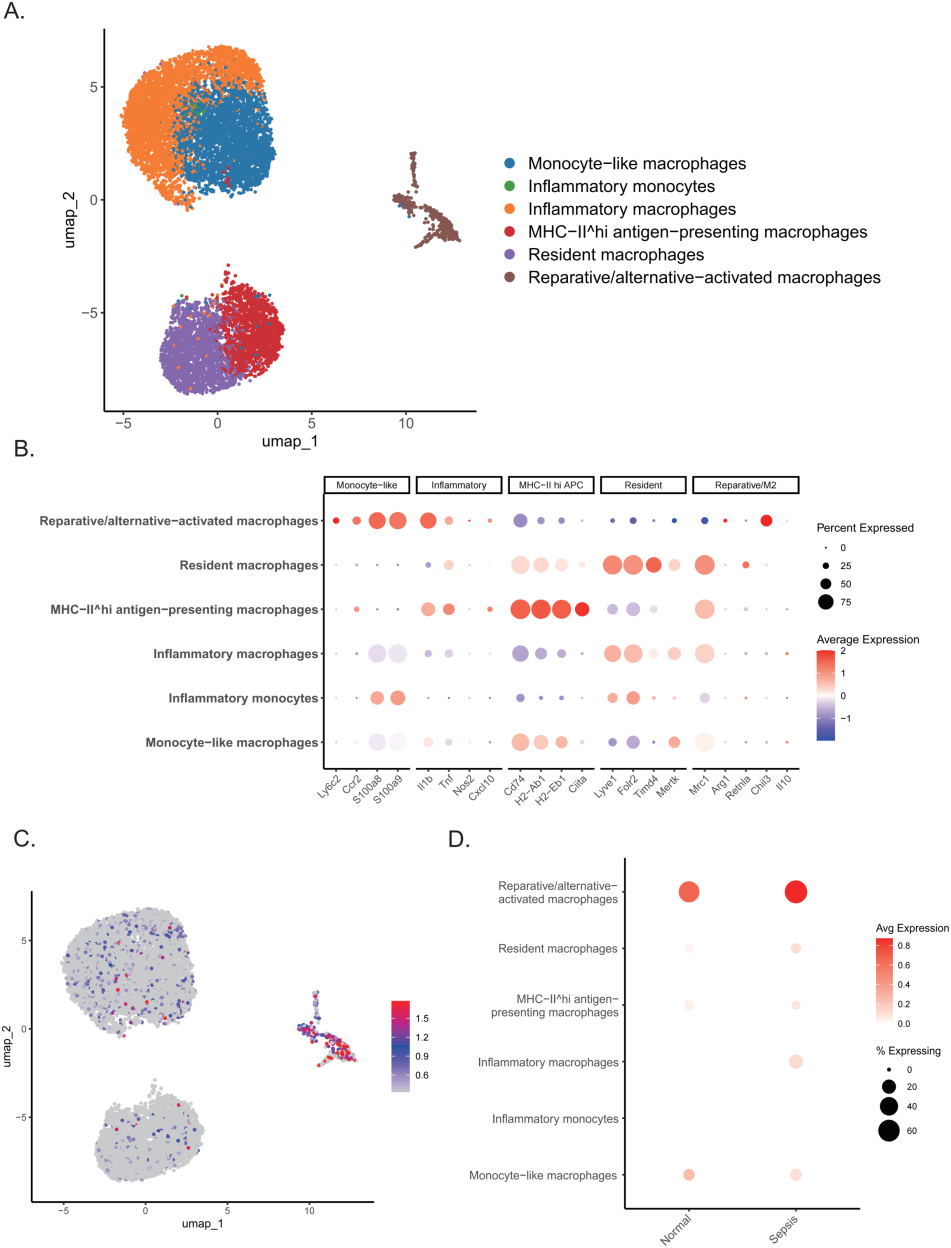
Single-cell analysis identifies macrophage heterogeneity and *Nfil3* expression patterns. **(A)** UMAP visualization showing distinct macrophage subclusters. **(B)** Dot plots showing representative marker genes used to define macrophage subsets. **(C)** Feature plot showing the distribution of *Nfil3* expression across macrophage populations. **(D)** Dot plot showing *Nfil3* expression levels in different macrophage subsets between sham and septic cardiomyopathy groups.

To further dissect macrophage heterogeneity and define the distribution of *Nfil3* at higher resolution, macrophage populations were extracted from the single-cell dataset and re-clustered. Unsupervised clustering identified multiple macrophage subsets, including monocyte-like macrophages, inflammatory monocytes, inflammatory macrophages, MHC-II^hi^ antigen-presenting macrophages, resident macrophages, and reparative/alternative-activated macrophages (**Figure 9A**). The identities of these subsets were confirmed using representative marker genes (**Figure 9B**). Feature plot analysis revealed that *Nfil3* expression was unevenly distributed across macrophage populations and was preferentially enriched in specific subsets (**Figure 9C**). Notably, *Nfil3* expression was markedly increased in inflammatory and reparative macrophage subsets under septic conditions. Consistently, dot plot analysis demonstrated both an increased proportion of *Nfil3*-expressing cells and elevated expression levels in these subsets in the septic cardiomyopathy group compared with sham controls (**Figure 9D**). Together, these findings support a macrophage-enriched and subset-specific upregulation of *Nfil3* in septic myocardium.

### CLP induces myocardial injury, cardiac dysfunction, and apoptosis in mice

**Figure 10:**
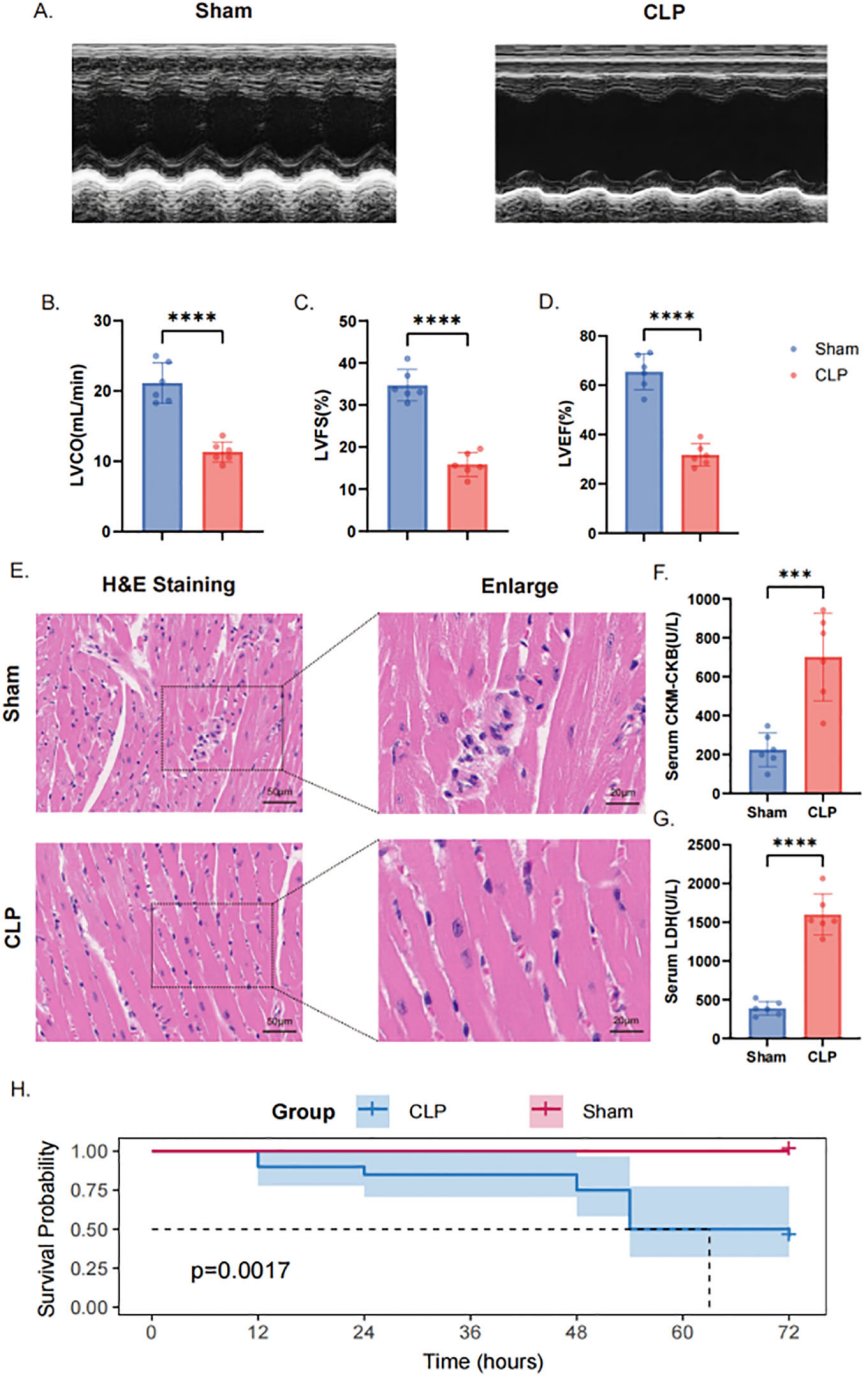
CLP induces myocardial injury and cardiac dysfunction in mice. **(A)** Representative echocardiographic images of sham-operated mice and CLP–induced septic mice. **(B–D)** Quantitative assessment of cardiac function: **(B)** left ventricular cardiac output (LVCO), **(C)** left ventricular fractional shortening (LVFS), and **(D)** left ventricular ejection fraction (LVEF) (n = 6 mice per group). **(E)** Representative hematoxylin and eosin (H&E)–stained myocardial sections (scale bar = 50 μm). **(F, G)** Serum levels of myocardial injury biomarkers: **(F)** creatine kinase-MB (CK-MB) and **(G)** lactate dehydrogenase (LDH) (n = 6 mice per group). **(H)** Kaplan–Meier survival curves (sham n = 20; CLP n = 20). Data are presented as mean ± SEM. ****P < 0.0001 versus the sham group.

A mouse model of septic cardiomyopathy was established using cecal ligation and puncture (CLP) to evaluate cardiac injury under septic conditions. Compared with sham-operated mice, CLP mice exhibited marked impairment of cardiac systolic function, as reflected by significant reductions in left ventricular cardiac output (LVCO), fractional shortening (LVFS), and ejection fraction (LVEF) (**Figures 10A–D**). Histopathological examination revealed disrupted myocardial architecture, pronounced inflammatory cell infiltration, and interstitial edema in CLP mice, whereas myocardial structure remained largely preserved in sham controls (**Figure 10E**). Consistent with these structural and functional abnormalities, serum levels of myocardial injury biomarkers, including creatine kinase-MB (CK-MB) and lactate dehydrogenase (LDH), were significantly elevated following CLP (**Figures 10F, G**). Survival analysis further demonstrated a substantially reduced 72-hour survival rate in CLP mice compared with sham controls (**Figure 10H**).

**Figure 11:**
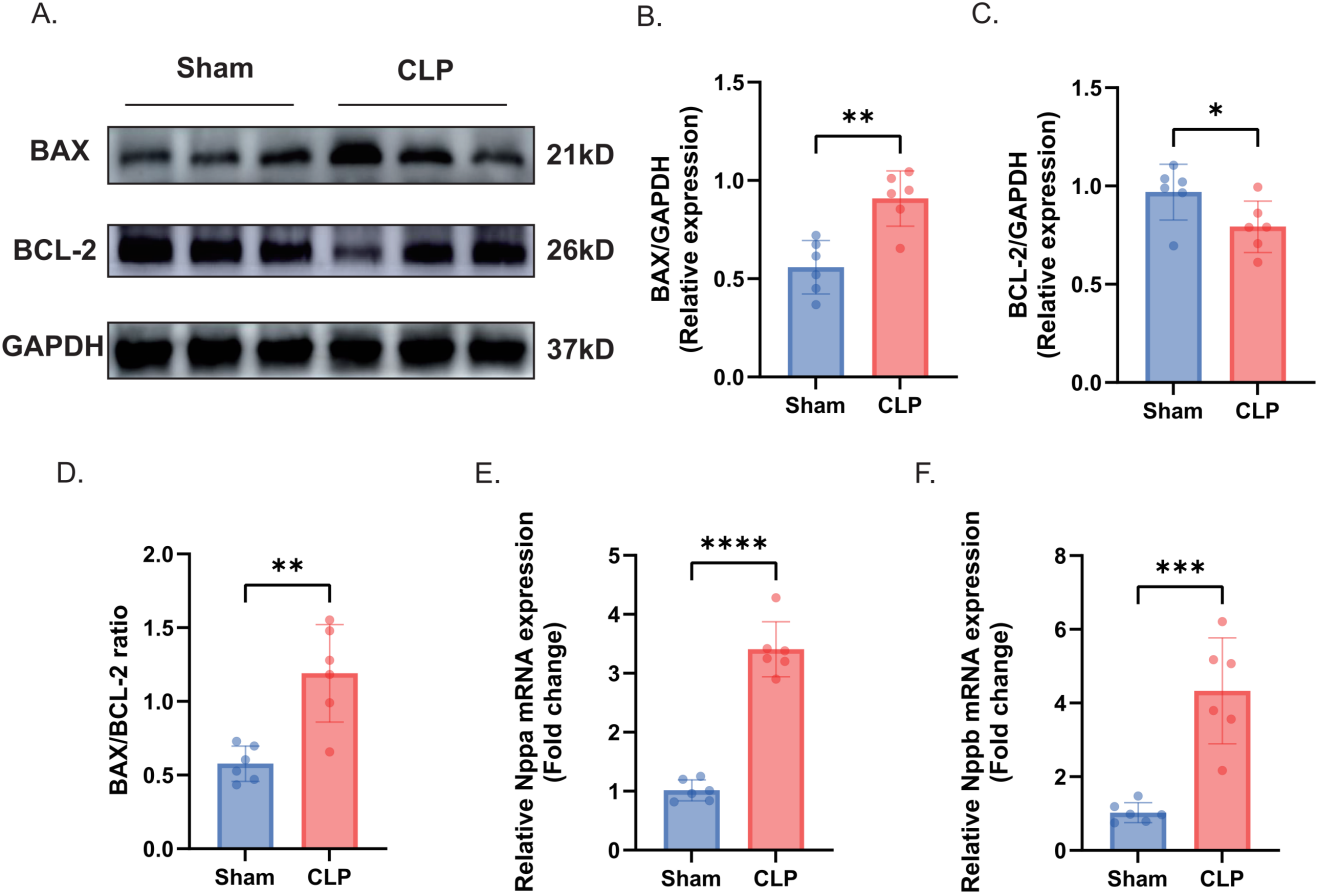
CLP-induced sepsis promotes myocardial injury and apoptosis. **(A)** Western blot analysis of apoptosis-related proteins BAX, BCL-2, and GAPDH in myocardial tissues from sham and CLP groups. **(B, C)** Quantification of BAX **(B)** and BCL-2 **(C)** protein expression normalized to GAPDH (n = 6 mice per group). **(D)** Quantification of the BAX/BCL-2 ratio in myocardial tissues (n = 6 mice per group). **(E, F)** qRT-PCR analysis of *Nppa* **(E)** and *Nppb* **(F)** mRNA expression levels (n = 6 independent samples per group). Data are presented as mean ± SEM. *P < 0.05, **P < 0.01, ***P < 0.001, ****P < 0.0001 versus the sham group.

Apoptosis-related protein expression was further assessed in myocardial tissues to characterize molecular alterations associated with myocardial injury. Western blot analysis demonstrated a significant increase in the pro-apoptotic protein BAX and a concomitant decrease in the anti-apoptotic protein BCL-2 in CLP mice compared with sham controls (**Figures 11A–C**). As a result, the BAX/BCL-2 ratio was markedly elevated, indicating a shift toward a pro-apoptotic state in septic myocardium (**Figure 11D**). In addition, qRT-PCR analysis showed significant upregulation of cardiac stress and injury markers, including *Nppa* and *Nppb*, in CLP mice (**Figures 11E, F**), further confirming myocardial damage at the transcriptional level. Together, these results demonstrate that CLP-induced sepsis leads to severe myocardial injury characterized by cardiac dysfunction, histopathological damage, and enhanced apoptosis.

### NFIL3 is upregulated in myocardial macrophages and attenuates inflammation by inhibiting NF-κB signaling

**Figure 12:**
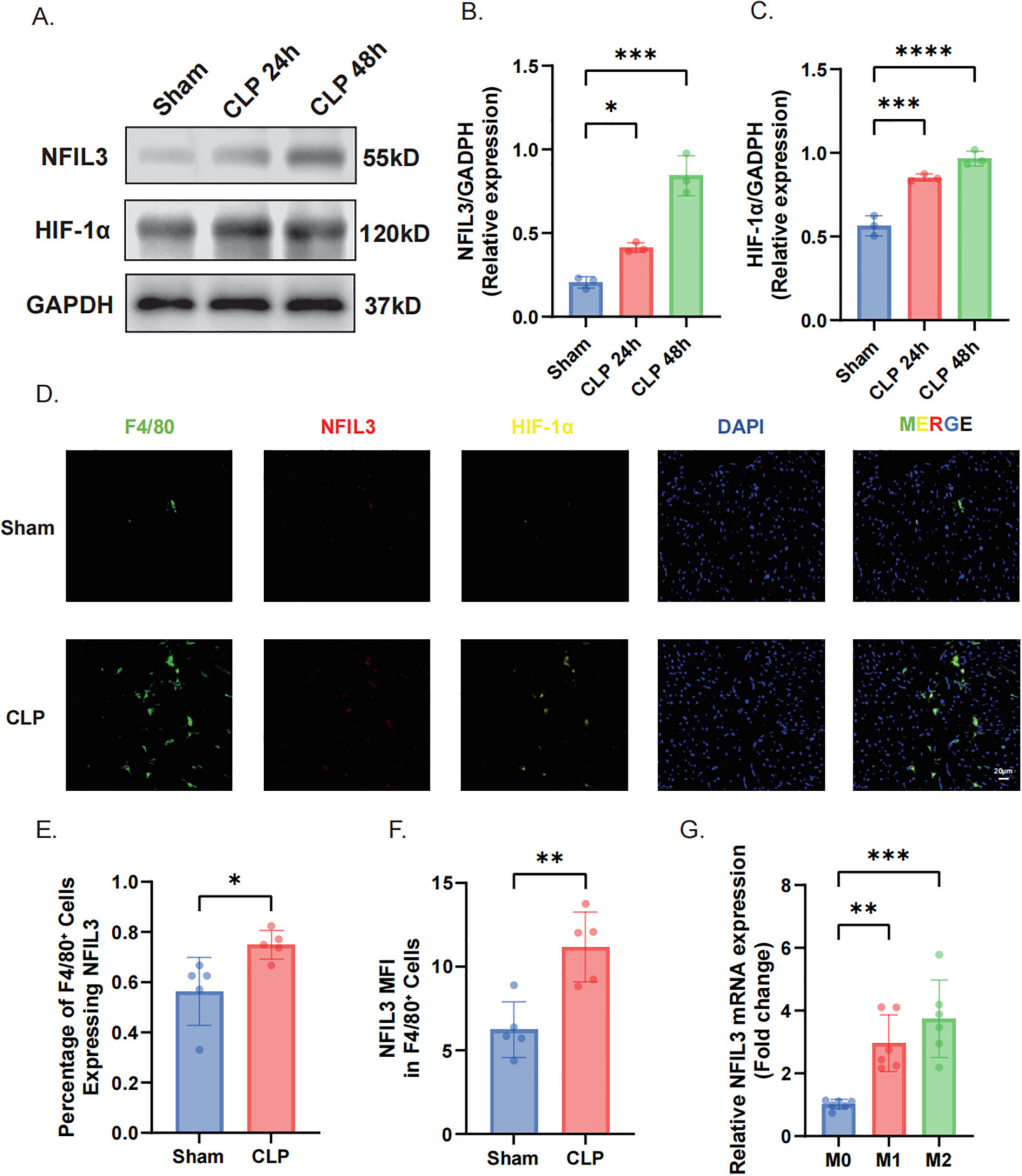
NFIL3 is upregulated in septic myocardium and preferentially expressed in macrophages. **(A)** Western blot analysis of NFIL3 and HIF-1α protein expression in myocardial tissue from sham, CLP 24 h, and CLP 48 h groups. **(B, C)** Quantification of NFIL3 **(B)** and HIF-1α **(C)** protein levels relative to the sham group (n = 3 mice per group). **(D)** Immunofluorescence staining of myocardial sections. Green: macrophage marker F4/80; red: NFIL3; yellow: HIF-1α; blue: DAPI. Scale bar = 50 μm. **(E)** Quantification of the proportion of F4/80-positive macrophages expressing NFIL3 in myocardial tissue (n = 5 mice per group). **(F)** Quantification of NFIL3 mean fluorescence intensity (MFI) in F4/80-positive macrophages (n = 5 mice per group). **(G)** qRT-PCR analysis of *Nfil3* mRNA expression in M1 (LPS + IFN-γ) and M2 (IL-4) macrophages (n = 6 independent experiments). Data are presented as mean ± SEM. *P < 0.05, **P < 0.01, ***P < 0.001, ****P < 0.0001 versus the sham group.

**Figure 13:**
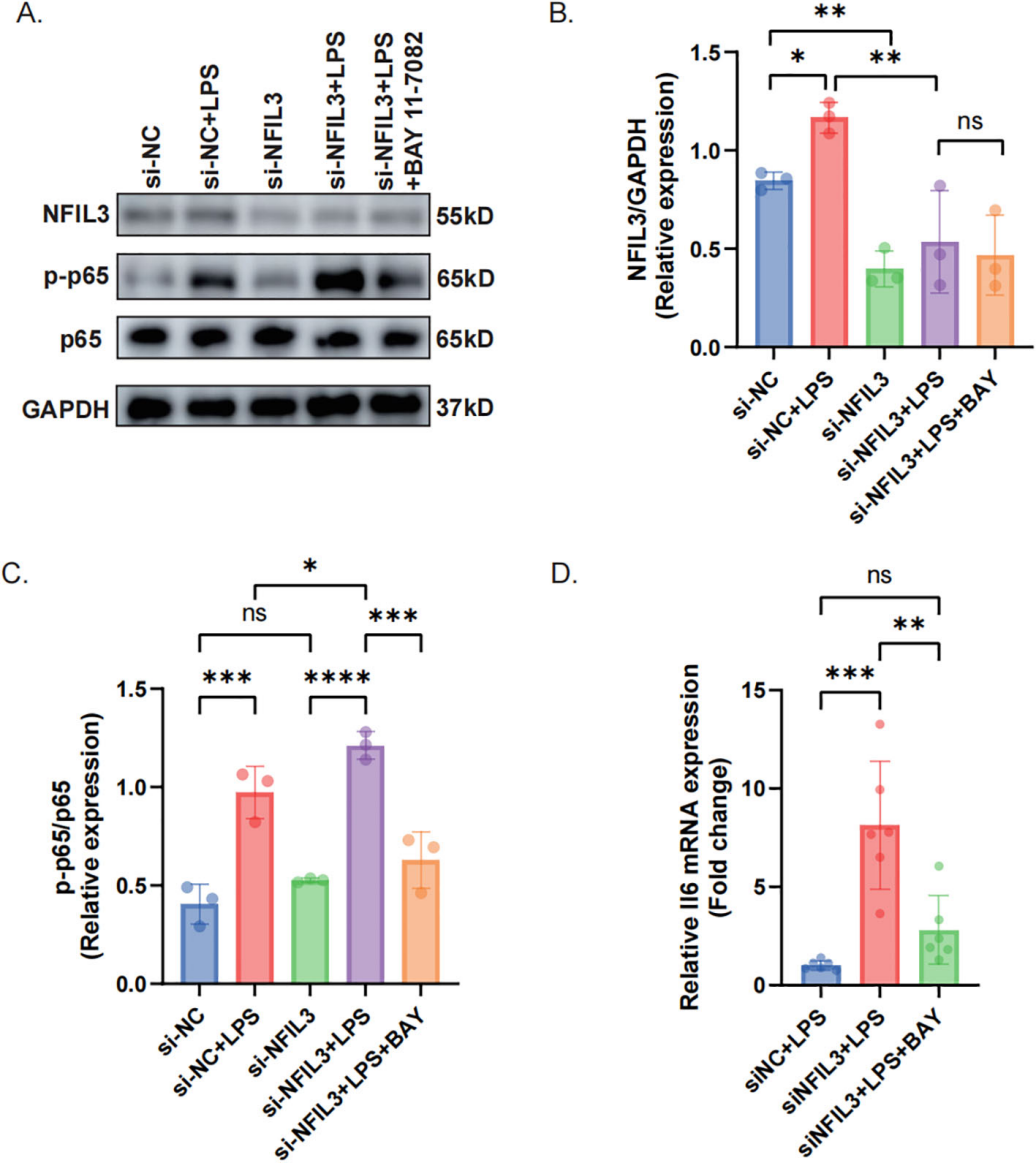
NFIL3 suppresses inflammation by inhibiting NF-κB signaling. **(A)** Western blot analysis of NFIL3, phosphorylated p65 (p-p65), total p65, and GAPDH protein expression in cells transfected with si-NC or si-Nfil3 and treated with LPS in the presence or absence of the NF-κB inhibitor BAY 11-7082. **(B, C)** Quantification of NFIL3 protein expression **(B)** and p-p65/p65 ratio **(C)** normalized to GAPDH (n = 3 independent experiments). **(D)** qRT-PCR analysis of *Il6* mRNA expression under the indicated conditions (n = 6 independent experiments). Data are presented as mean ± SEM. *P < 0.05, **P < 0.01, ***P < 0.001, ****P < 0.0001; ns, not significant.

To characterize NFIL3 expression dynamics during septic cardiomyopathy, myocardial tissues from CLP and sham controls were analyzed at different time points. Western blot analysis revealed a significant and time-dependent increase in NFIL3 protein expression at 24 and 48 hours after CLP compared with sham controls. In parallel, the hypoxia-responsive transcription factor HIF-1α was also markedly upregulated, indicating activation of hypoxic signaling in septic myocardium (**Figures 12A–C**). Immunofluorescence staining further demonstrated prominent localization of NFIL3 within F4/80-positive macrophages in CLP mice (**Figure 12D**). Quantitative analyses confirmed that both the proportion of NFIL3-expressing macrophages and the intracellular NFIL3 mean fluorescence intensity were significantly increased in septic myocardium compared with sham controls (**Figures 12E, F**). Consistent with the *in vivo* observations, qRT-PCR analysis showed increased *Nfil3* mRNA expression in both M1-polarized (LPS + IFN-γ) and M2-polarized (IL-4) macrophages *in vitro*, with higher expression levels observed in M2 macrophages (**Figure 12G**). Together, these results demonstrate that NFIL3 is persistently upregulated at both transcriptional and protein levels during septic cardiomyopathy and is preferentially expressed in myocardial macrophages under hypoxic and inflammatory conditions. Given its marked enrichment in macrophages, we next sought to investigate its functional role in regulating inflammatory signaling. *Nfil3* expression was silenced in macrophages followed by LPS stimulation. Western blot analysis showed that *Nfil3* knockdown significantly enhanced NF-κB activation, as indicated by increased phosphorylation of p65, while treatment with the NF-κB inhibitor BAY 11–7082 effectively attenuated this effect (**Figures 13A–C**). Consistently, qRT-PCR analysis demonstrated that *Nfil3* deficiency markedly increased *Il6* mRNA expression, whereas pharmacological inhibition of NF-κB signaling significantly reduced *Il6* levels (**Figure 13D**). These findings indicate that NFIL3 negatively regulates macrophage inflammatory responses, potentially through inhibition of NF-κB signaling.

## Discussion

Septic cardiomyopathy (SCM) is a major contributor to the elevated mortality observed in patients with sepsis (27, 28). Although substantial progress has been made in elucidating its pathophysiological mechanisms, the underlying molecular regulatory network remains incompletely understood, particularly the crosstalk between hypoxic stress and immune dysregulation (29, 30). In this study, we integrated transcriptomic analysis, single-cell sequencing, machine learning–based modeling, Mendelian randomization, and *in vivo* and *in vitro* validation to achieve a multi-level, systems-level characterization of the molecular landscape of septic cardiomyopathy. Our findings suggest that hypoxia-driven immune remodeling—especially macrophage-centered inflammatory reprogramming—plays a pivotal role in disease progression. Moreover, NFIL3 was identified as a key molecular hub linking hypoxic stress, immune regulation, and myocardial dysfunction.

Accumulating evidence indicates that septic cardiomyopathy does not result solely from direct pathogen-mediated myocardial damage, but rather from the combined effects of host metabolic dysregulation and immune imbalance. Previous studies have shown that sepsis induces functional tissue hypoxia through mechanisms such as microcirculatory dysfunction, mitochondrial impairment, and reduced oxygen utilization efficiency (31, 32). In lipopolysaccharide (LPS)- or cecal ligation and puncture (CLP)-induced sepsis models, hypoxia-responsive factors, including hypoxia-inducible factor-1α (HIF-1α), are markedly activated. These factors exacerbate myocardial energy deficiency and contractile dysfunction by suppressing mitochondrial oxidative phosphorylation and promoting glycolytic metabolic reprogramming. Notably, HIF-1α has been shown to be upregulated in septic myocardium via NF-κB–mediated signaling, leading to impaired mitochondrial respiration and subsequent deterioration of myocardial contractile and metabolic function (33).

Consistent with these mechanisms, gene set enrichment analysis revealed significant enrichment of hypoxia-related and multiple metabolic pathways in septic myocardium. Intersection analysis of differentially expressed genes with the HALLMARK_HYPOXIA gene set identified a core group of hypoxia-associated genes. Functional annotation indicated that these genes are predominantly involved in nitric oxide signaling, cellular stress responses, and tissue repair processes. Collectively, these findings suggest that hypoxia in septic myocardium is not merely a consequence of energy imbalance but functions as a critical upstream regulator of metabolic reprogramming, immune cell activity, and inflammatory responses.

Among diverse immune cell populations, macrophages represent one of the most critical drivers of immune dysregulation in septic cardiomyopathy, contributing substantially to both inflammatory amplification and myocardial injury. Immune infiltration analysis revealed a shift toward a disrupted balance between pro-inflammatory and anti-inflammatory responses in the septic myocardium. These observations are highly consistent with previous studies highlighting macrophage heterogeneity in septic cardiomyopathy. Accumulating evidence indicates that pro-inflammatory M1 macrophages aggravate myocardial injury through multiple mechanisms, including excessive cytokine release, mitochondrial damage, and the induction of ferroptosis (34). In contrast, specific cardiac resident macrophage subpopulations, such as TREM2^hi^ resident macrophages, confer cardioprotective effects by facilitating the clearance of damaged mitochondria and preserving myocardial homeostasis (35).

The functional states of distinct macrophage subpopulations are tightly regulated by multiple signaling pathways. For instance, activation of the Notch1 pathway has been shown to exacerbate inflammation and myocardial injury by suppressing mitochondrial autophagy and promoting NLRP3 inflammasome activation (36). Meanwhile, HIF-1α is increasingly recognized as a central molecular link between hypoxic stress and macrophage inflammatory polarization. Previous studies have demonstrated that recombinant thrombomodulin (rTM) improves survival in septic mice by modulating the HIF-1α/METTL3/PFKM axis, thereby inhibiting macrophage glycolysis and reducing the production of proinflammatory cytokines, including IL-1β, IL-6, and TNF-α (37). In the context of septic cardiomyopathy, the HIF-1α/NF-κB signaling axis has been identified as a key driver of macrophage inflammatory activation and cardiac dysfunction (33). Notably, this mechanism has been validated across multiple chronic inflammatory disease models and is frequently accompanied by metabolic reprogramming characterized by HIF-1α–dependent enhancement of glycolysis, which is essential for sustaining the proinflammatory phenotype and cell survival (38, 39).

Against this background, we constructed a diagnostic model comprising NFIL3, SDC4, and TGM2 across multiple independent human cohorts through protein–protein interaction network analysis and cross-validation using diverse machine learning algorithms. Among these genes, SDC4, a transmembrane heparan sulfate proteoglycan, plays a critical role in cell adhesion, migration, and inflammatory signaling, with dysregulated expression closely associated with tissue fibrosis and injury repair processes (40–42). TGM2, an enzyme involved in protein cross-linking and extracellular matrix stabilization, has been widely reported to be upregulated under conditions of cellular stress, apoptosis, and fibrotic remodeling (43, 44). Importantly, subsequent single-gene model validation and mechanistic analyses revealed that NFIL3 exhibited the most robust and stable diagnostic performance, as well as prominent characteristics of a central biological regulatory hub.

NFIL3 (also known as E4BP4) is a ubiquitously expressed basic leucine zipper (bZIP) transcription factor that plays diverse roles in immune cell differentiation, innate lymphoid cell development, inflammatory regulation, and cell survival. Its essential function in innate lymphoid cell (ILC) differentiation and functional maintenance is well established (45, 46), and our previous work demonstrated a protective role of ILC2s in the septic heart (6). However, the biological functions of NFIL3 are highly context dependent. Recent evidence suggests that under cellular stress conditions, nuclear stress bodies selectively enhance NFIL3 transcription through three-dimensional genome remodeling, thereby suppressing the expression of key inflammatory mediators in macrophages (47). Conversely, in sepsis-associated acute kidney injury, NFIL3 has been reported to promote ferroptosis and inflammatory responses by regulating lipid metabolic pathways (48), underscoring the strong dependence of its function on cell type and pathological microenvironment.

Single-cell transcriptomic analysis further supports these observations at the cellular level. In septic cardiomyopathy, NFIL3 was markedly upregulated in both cardiac tissue and peripheral blood, with predominant enrichment in myocardial macrophage populations. Concurrently, AUCell analysis demonstrated significantly elevated hypoxia scores across the global cell population and multiple immune cell subsets. Collectively, these findings suggest that within the hypoxic myocardial microenvironment, NFIL3 may function as a critical transcriptional regulatory node linking hypoxic stress to immune inflammatory responses, potentially operating through mechanisms distinct from classical single-pathway inflammatory signaling.

In addition, our *in vitro* polarization experiments further demonstrated that NFIL3 expression is upregulated in both M1 and M2 macrophages, with higher levels observed in M2-polarized cells, indicating that NFIL3 is dynamically regulated rather than restricted to a specific polarization state. This dynamic expression pattern is consistent with previous studies showing that NFIL3 can be induced by inflammatory stimuli such as LPS and functions as a transcriptional regulator that restrains excessive inflammatory responses in macrophages (49). Moreover, recent evidence indicates that NFIL3 is associated with anti-inflammatory gene programs and macrophage phenotypic regulation (50). Importantly, our findings extend previous observations by demonstrating that NFIL3 modulates NF-κB signaling in macrophages under septic conditions. Given that NF-κB is a central driver of inflammatory amplification in sepsis, this regulatory axis may represent a critical checkpoint linking hypoxia-induced transcriptional responses to downstream inflammatory cascades.

Through Mendelian randomization (MR) analysis, we observed a positive association between genetically predicted NFIL3 expression and the risk of sepsis. At first glance, this observation appears discordant with the anti-inflammatory effects of NFIL3 reported in certain experimental settings. However, these findings likely reflect differences in the biological levels and disease stages captured by each approach.

Specifically, Mendelian randomization estimates the lifelong effect of genetically determined gene expression on disease susceptibility, rather than the dynamic, stage-dependent functions of genes during acute disease progression (51). In this context, individuals with higher baseline NFIL3 expression may be predisposed to immune tolerance or immunosuppressive states, thereby compromising early pathogen clearance and increasing susceptibility to severe infection or sepsis (52, 53).

Accordingly, the upregulation of NFIL3 observed in sepsis patients and experimental models is more likely indicative of a compensatory response aimed at restraining excessive inflammation, rather than a primary driver of disease initiation. This distinction between genetic susceptibility and disease-stage-specific function is well recognized in immunological research and highlights that NFIL3 does not function as a simple anti-inflammatory protective factor. Instead, NFIL3 acts as an immune negative regulatory node that becomes aberrantly activated under hypoxic conditions, and its sustained upregulation may ultimately contribute to immune imbalance (51, 54).

In both the CLP model and LPS-stimulated *in vitro* experiments, we further observed synchronous upregulation and distinct spatial colocalization of NFIL3 and the core hypoxia-responsive transcription factor HIF-1α in cardiac macrophages. These findings strongly link NFIL3 expression to the hypoxic microenvironment of the septic myocardium. Previous studies have elucidated upstream regulatory mechanisms governing NFIL3 expression in immune cells. For example, in cytotoxic T cells, IL-2–JAK1/3 signaling induces NFIL3 expression by stabilizing HIF-1α (55), whereas hypoxic stimulation alone is sufficient to markedly increase NFIL3 protein levels through HIF-1α–dependent transcriptional programs (56). Moreover, Yu et al. proposed that under hypoxic conditions, HIF-1α directly induces NFIL3 expression and establishes a “HIF-1α–NFIL3–PIM1” regulatory axis with its downstream effector PIM1, thereby shaping cellular adaptation and fate decisions in response to hypoxic stress (57).

Based on these lines of evidence, we hypothesize that within the hypoxic microenvironment of septic myocardium, NFIL3 may function as a downstream effector of HIF-1α signaling. Through its involvement in macrophage metabolic reprogramming, polarization shifts, and inflammatory phenotype remodeling, NFIL3 may play a crucial regulatory role in the progression of septic cardiomyopathy. Notably, given the observed co-upregulation and colocalization of NFIL3 with HIF-1α, it is plausible that NFIL3 serves as a molecular link connecting hypoxia signaling to downstream inflammatory regulation. Our *in vitro* experiments further demonstrate that NFIL3 suppresses macrophage inflammatory responses, at least in part, by inhibiting NF-κB signaling activation, as evidenced by increased p65 phosphorylation upon NFIL3 knockdown and its reversal by pharmacological inhibition. This finding is consistent with previous studies indicating that NFIL3 functions as a transcriptional repressor of pro-inflammatory signaling pathways, further supporting its role as a negative regulator of inflammation.

From a clinical translational perspective, the acquisition of consecutive myocardial tissue samples from patients with sepsis is impractical. To address this limitation, we performed a cross-species integrative analysis by mapping key hypoxia-related genes identified in mouse myocardium to human peripheral blood transcriptomic datasets. This approach revealed that NFIL3 exhibits stable diagnostic performance in peripheral blood, suggesting its potential utility as a circulating biomarker reflecting hypoxia-driven immune remodeling within the myocardium. Importantly, NFIL3 should not be interpreted as a classical protective factor; rather, it appears to function as an aberrantly activated immune negative regulatory node in sepsis, whose sustained upregulation may further aggravate immune dysregulation.

Several limitations of this study should be acknowledged. First, although multiple population-based datasets and experimental models were integrated, the clinical diagnostic and prognostic value of NFIL3 requires further validation in large, prospective cohorts. In addition, although our data suggest that NFIL3 regulates inflammatory responses via the NF-κB pathway, the precise molecular interactions and upstream regulatory mechanisms require further investigation.

## Conclusion

In conclusion, the hypoxia pathway plays a pivotal role in the immunoregulatory mechanisms underlying sepsis-induced myocardial injury. By integrating multi-omics data with machine learning approaches, we identified NFIL3, SDC4, and TGM2 as key hub genes within this pathway. A diagnostic model constructed based on these three genes exhibited excellent predictive performance across multiple independent cohorts, highlighting its potential utility for the early diagnosis and dynamic monitoring of septic myocardial injury. To our knowledge, this study provides integrative evidence supporting a role for NFIL3 in hypoxia-driven immune remodeling in SCM. NFIL3 may serve as a biomarker and a candidate therapeutic target, although further *in vivo* validation is required.

## Data Availability

The datasets analysed during the current study are available in the Gene Expression Omnibus (GEO) repository under accession numbers GSE229925, GSE267388, GSE65682, GSE134347, GSE66099, GSE79962 and GSE190856. The hypoxia-related gene set was sourced from the MSigDB database. Detailed descriptions of these data are provided in the Methods section.
