# Supplementary material for: Multi-omics analysis identifies NFIL3 as a hypoxia-associated immune regulator in septic cardiomyopathy
Source: Front Immunol. 2026 Apr 30;17:1785241. doi: 10.3389/fimmu.2026.1785241 (PMC13171352; doi:10.3389/fimmu.2026.1785241)
Supplement: Supplementary file 1 [file DataSheet1.docx]

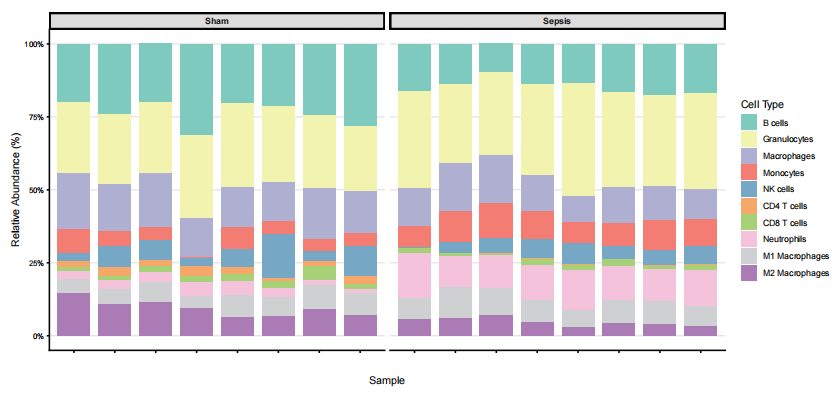


**Supplementary Figure S1. Composition of cardiac immune cells in sham and septic mice.** Stacked bar chart showing the relative proportions of major immune cell subsets (e.g., macrophages, T cells, neutrophils, B cells, etc.) in cardiac tissue of sham control and sepsis model mice.


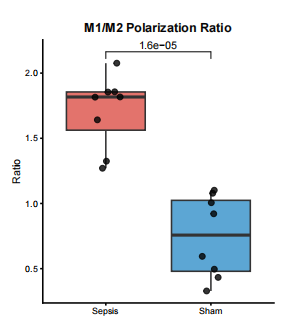


**Supplementary Figure S2. Alteration of macrophage polarization in septic cardiomyopathy.** Box plot comparing the M1/M2 macrophage ratio between sham and sepsis groups. Data are presented as the mean ± SEM. Statistical significance was determined by Student’s t-test; exact p value is shown on the graph.


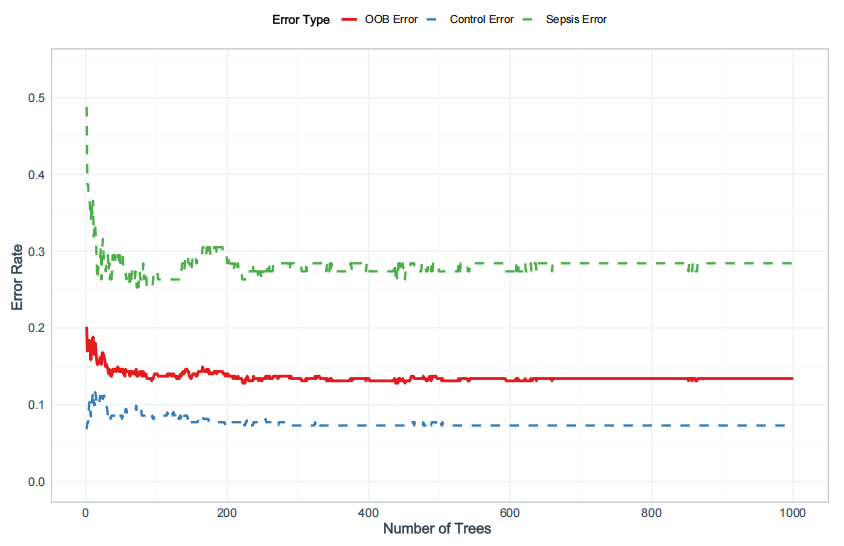


**Supplementary Figure S3. Tuning curve of the random forest model.** Line graph showing the relationship between the number of decision trees in the forest and the corresponding out-of-bag (OOB) error rate. The point at which the error rate stabilizes indicates a sufficient number of trees for a robust model.


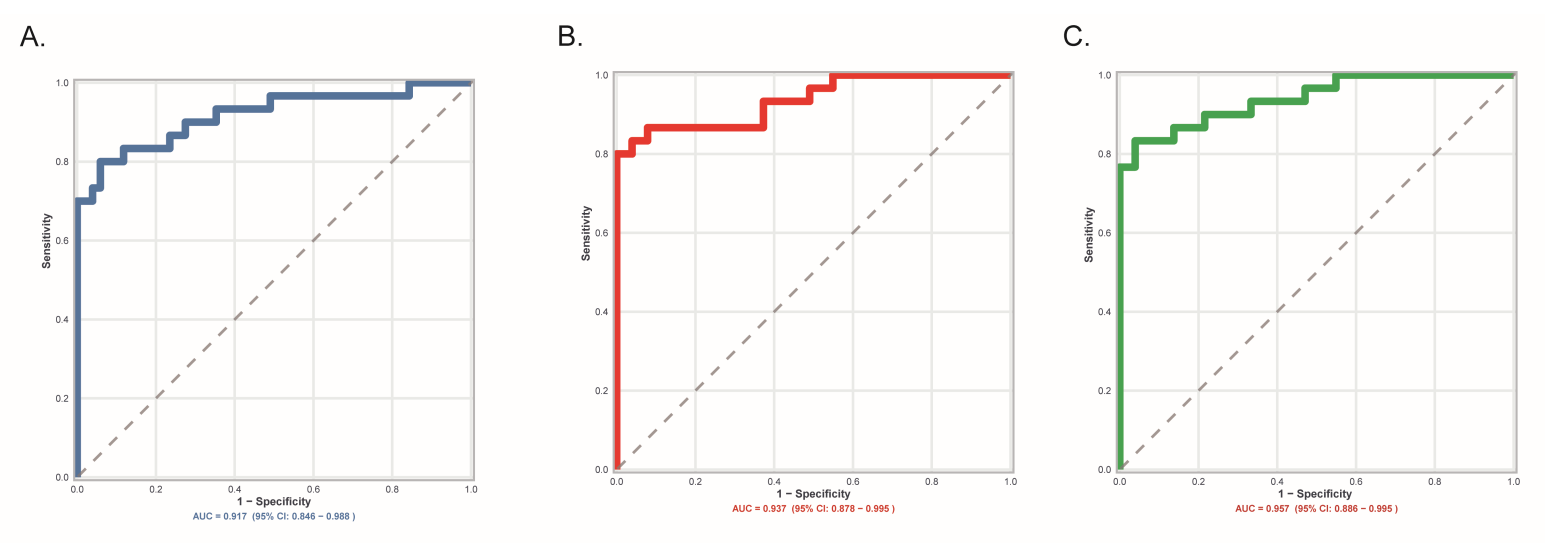


**Supplementary Figure S4. Receiver operating characteristic (ROC) curves of diagnostic models. ROC curves comparing the diagnostic performance of biomarker panels identified by three distinct machine learning algorithms: (A) Least Absolute Shrinkage and Selection Operator (LASSO) regression, (B) Random Forest, and (C) Support Vector Machine - Recursive Feature Elimination (SVM-RFE).** The area under the curve (AUC) with 95% confidence interval is displayed for each model.


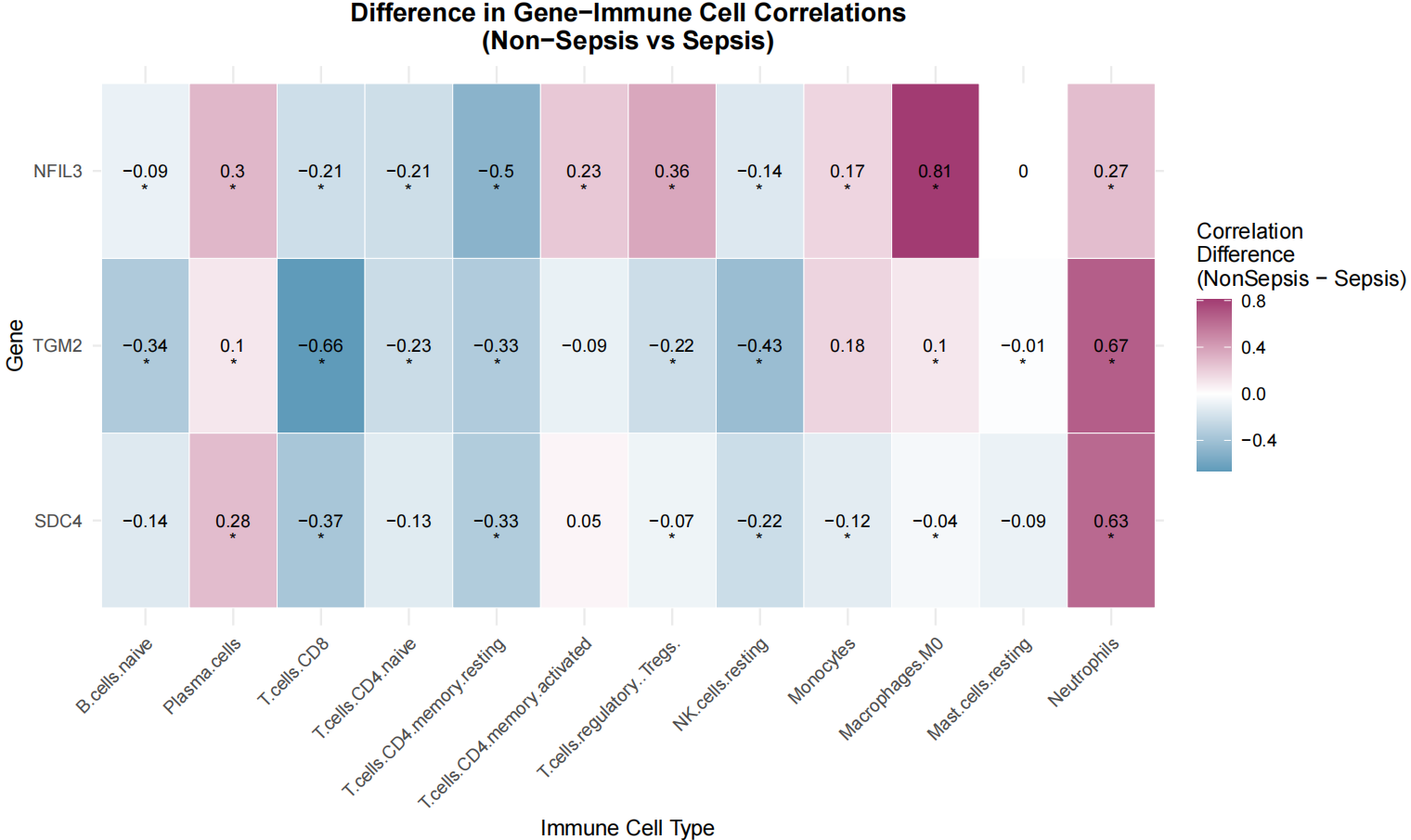


**Supplementary Figure S5. Comparative gene expression profiles in immune cells between non-sepsis and sepsis groups.** Heatmap of the expression levels of NFIL3, TGM2, and SDC4 across major immune cell types. Data are presented for both non-sepsis and sepsis groups. Statistical significance is denoted by asterisks (**P* < 0.05).
