# Supplementary material for: Multi-omics analysis identifies NFIL3 as a hypoxia-associated immune regulator in septic cardiomyopathy
Source: Front Immunol. 2026 Apr 30;17:1785241. doi: 10.3389/fimmu.2026.1785241 (PMC13171352; doi:10.3389/fimmu.2026.1785241)
Supplement: Supplementary file 2 [file DataSheet2.docx]

**Supplementary Table S1. Summary of transcriptomic datasets and hypoxia-related gene sets used in this study**

| **Dataset** | **Database** | **Platform** | **Sample Description** |
| --- | --- | --- | --- |
| GSE229925 | GEO | GPL24247 | Mouse myocardial tissue, sepsis model classified by left ventricular ejection fraction (LVEF) |
| GSE267388 | GEO | GPL28330 | Mouse model of sepsis-induced myocardial dysfunction |
| GSE65682 | GEO | GPL570 | Whole blood leukocyte transcriptomes from critically ill patients (including sepsis) and healthy controls |
| GSE134347 | GEO | GPL17586 | Whole blood leukocyte transcriptomes from sepsis patients, non-infectious critically ill patients, and healthy controls |
| GSE66099 | GEO | GPL570 | Whole blood samples from pediatric patients with SIRS, sepsis/septic shock, and healthy controls |
| GSE79962 | GEO | GPL6244 | Cardiac tissue from sepsis non-survivors and non-failing donor hearts |
| GSE190856 | GEO | GPL24247 | Single-cell RNA sequencing atlas of cardiac tissue from a mouse model of septic cardiomyopathy and control mice |
| Hypoxia Gene Set | MSigDB | HALLMARK Gene Sets | HALLMARK_HYPOXIA gene set, used for hypoxia pathway enrichment analysis |
